# *Bacteroides Fragilis* Exacerbates T2D Vascular Calcification by Secreting Extracellular Vesicles to Induce M2 Macrophages

**DOI:** 10.1002/advs.202410495

**Published:** 2024-12-12

**Authors:** Cong Chen, Zhengfeng Liang, Yuqi He, Yan Gao, Shuhui Ouyang, Lili Wang, Jianghua Liu, Jingsong Cao

**Affiliations:** ^1^ The First Affiliated Hospital Department of Laboratory Medicine Hengyang Medical School University of South China Hengyang Hunan 421001 China; ^2^ School of Pharmaceutical Science Hengyang Medical School University of South China Hengyang Hunan 421001 China; ^3^ The First Affiliated Hospital Institute of Endocrinology and metabolism Center for Clinical Research in Diabetes Hengyang Medical School University of South China Hengyang Hunan 421001 China

**Keywords:** *Bacteroides fragilis*, extracellular vesicles, macrophages, type 2 diabetes, vascular calcification

## Abstract

Vascular calcification (VC) in type 2 diabetes (T2D) poses a serious threat to the life and health of patients. However, its pathogenesis remains unclear, resulting in a lack of effective treatment for the root cause. It is found that both intestinal *Bacteroides fragilis* (BF) and peripheral M2 monocytes/macrophages are significantly elevated in patients with T2D VC. M2 macrophages are identified as a significant risk factor for T2D VC. Both BF and their extracellular vesicles (EV) promote T2D VC and facilitate macrophage M2 polarization. Macrophages clearance significantly antagonized BF EV‐induced T2D VC in mice. Mechanistically, EV‐rich double‐stranded DNA (dsDNA) activates stimulator of interferon response cGAMP interactor 1 (Sting), promotes myocyte enhancer factor 2D (Mef2d) phosphorylation, upregulates tribbles pseudokinase 1 (Trib1) expression, and induces macrophage M2 polarization. Concurrently, Mef2d activated by the EV targets and upregulates the expression of pro‐calcification factor Serpine1, thereby exacerbating T2D VC. Clinical studies have shown that Serpine1 is significantly elevated in the peripheral blood of patients with T2D VC and is closely associated with T2D VC. In summary, this study reveals that intestinal BF promotes Trib1 expression through the EV‐Sting‐Mef2d pathway to induce macrophage M2 polarization and upregulates serpin family E member 1 (Serpine1) expression, thereby aggravating T2D VC. The findings provide a new theoretical and experimental bases for optimizing the strategies for prevention and treatment of T2D VC.

## Graphical abstract

*Bacteroides fragilis*, an intestinal bacterium associated with T2D VC, secretes extracellular vesicles that activate the Sting‐Mef2d‐Trib1 signaling pathway, promoting macrophage M2 polarization. Additionally, Extracellular vesicles activates the Sting‐Mef2d axis, which enhances the expression and secretion of Serpine1, thereby facilitating VC. Notably, both M2 macrophages and Serpine1 levels in serum show a positive correlation with T2D VC.

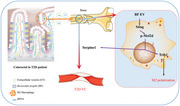

## 1 Introduction

Recently, patients with T2D have experienced a higher incidence and severer degree of VC, which results in an increase in the incidence and mortality of chronic vascular complications in patients with T2D. However, the underlying mechanisms of VC remain poorly understood.^[^ ^1^, ^2^ ^]^ Considering that VC has important clinical consequences, identifying its initiating mechanisms is crucial to devising strategies to prevent, inhibit, or dislodge calcium deposits from the arterial wall.

VC is characterized by the ectopic mineralization of calcium ions along blood vessels, representing a pathological process of mineralization that occurrs within the blood vessel wall.^[^ ^3^, ^4^, ^5^ ^]^ Runx2, a key initiating gene for the initiation of osteogenic differentiation in vascular smooth muscle cells (VSMCs), exhibits significant upregulation,^[^ ^6^ ^]^ which is recognized as an important marker of this differentiation process.^[^ ^7^, ^8^ ^]^ Previouse reasearch indicates that alterations in food structure and eating habits contribute to the high incidence of diabetes and related vascular complications, as well as to affects the composition of intestinal flora.^[^ ^9^, ^10^, ^11^ ^]^ Thus, intestinal bacteria have emerged as a focal point in the prevention and treatment of T2D and the vascular complications associated with it.^[^ ^12^, ^13^ ^]^

Intestinal microorganisms, often referred to as the “forgotten organ” and the “human second genome”,^[^ ^14^ ^]^ influence various organs including the intestine,^[^ ^15^ ^]^ bone marrow,^[^ ^16^ ^]^ brain,^[^ ^17^ ^]^ and liver^[^ ^18^ ^]^ by secreting secondary metabolites. In addition to secondary metabolites, vesicles represent another significant mechanism through which intestinal bacteria exert their biological effects.^[^ ^19^, ^20^, ^21^ ^]^ Tang W et al. observed that intestinal bacterial vesicles can induce M1 polarization of intestinal macrophages upon entering the body,^[^ ^22^ ^]^ and can also affect the metabolic activity of liver macrophages via the peripheral blood.^[^ ^23^ ^]^

Macrophages are immune cells that are widely distributed across various tissues and organs. They can polarize into two distinct phenotypes: the pro‐inflammatory M1 phenotype and anti‐inflammatory M2 phenotype.^[^ ^24^ ^]^ Recent single‐cell sequencing studies have identified macrophages as the predominant immune cell type in atherosclerotic lesions in both humans and mice.^[^ ^25^, ^26^ ^]^ Our previous research has demonstrated that a high‐glucose environment induces M1 polarization in macrophages, thereby promoting VC associated with T2D.^[^ ^27^, ^28^ ^]^ However, other studies indicate that M2 macrophages play a crucial role in stabilizing vascular plaques in T2D.^[^ ^29^ ^]^ This suggests that the excessive activation of macrophages contributes significantly to the progression of T2D VC.

Here, we first screened intestinal bacteria associated with T2D VC, and subsequently analyzed the causal relationship between the intestinal bacterium BF and macrophage polarization, demonstrating that BF induces macrophage M2 polarization at both in the cellular and animal. This research further elucidates the molecular mechanisms that promote T2D VC and reveals the correlation of M2 macrophages and cytokines with T2D VC. This work provide new theoretical foundations and experimental support for optimizing the strategies for prevention and control of T2D VC.

## 2 Experimental Section

### 2.1 Cell Culture

RAW264.7 and 293T cells were obtained from the American Type Culture Collection (ATCC) and cultured in high‐glucose Dulbecco's modified Eagle's medium (DMEM, Gibco BRL, Grand Island, USA) supplemented with 10% fetal bovine serum (FBS, Gibco, Australia) and 100 U ml^−1^ penicillin‐streptomycin at 37 °C in a 5% CO_2_ atmosphere. THP‐1 cells (ATCC) were cultured in 1640 medium (Gibco, Australia) with 10% FBS and 100 U ml^−1^ penicillin‐streptomycin at 37 °C with 5% CO_2_.

### 2.2 Separation and Culture of Mouse VSMCs

Aortas were harvested from 8‐10‐week‐old mice, the extravascular fat was removed, and the aortas were opened along the vessel lumen. The tunica adventitia and endothelium of the vascular wall were carefully dissected. The aortas were then cut into small pieces and placed in 1 ml of culture medium (DMEM with 10% FBS and 1% penicillin‐streptomycin), followed by centrifugation at 1000 rpm for 1 min. The resulting precipitate was resuspended in 2 ml of culture medium and transferred to a T25 bottle (2 mice per bottle). After overnight incubation at 37 °C and 5% CO_2_, an additional 2 ml of culture medium was added, with the medium changed every 2 days. Finally, the VSMCs were isolated for further cultivation or analysis.

### 2.3 Transwell Assay

The impact of macrophages on VSMCs was evaluated using Transwell plates (Corning Incorporated, NY, USA) with a 0.4 µm pore membrane. RAW264.7 cells were seeded in the upper chamber of 24‐well plates (1 × 10^5^ cells) or 6‐well plates (1 × 10^6^ cells). An equal number of VSMCs was seeded on the lower surface. After incubation at 37 °C with 5.0% CO_2_ for 48 h, VSMCs were harvested for further analysis. Alternatively, the RAW 264.7 cells in the upper chamber were initially exposed to 1 µg ml^−1^ BF EV for 24 h, washed thrice with the culture medium, and finally co‐cultured with the VSMCs at 37 °C with 5.0% CO_2_ for 48 h.

### 2.4 Patients Inclusion and Exclusion Criteria

Inclusion criteria: Participants aged 20–70 years were included if they had been diagnosed with T2D according to the American Diabetes Association standards for more than 6 months, had no systemic diseases other than T2D, and had not experienced any infections up to one month before enrollment. The participants were required to not be on a diet or any medication that could affect blood sugar homeostasis. Clinical diagnosis was based on the presence or absence of coronary calcification.

Exclusion criteria: Patients were excluded if they had any major systemic diseases such as tumors, clinically proven hemoglobinopathy or anemia, history of drug or alcohol abuse (defined as >80 g d^−1^ for males and >40 g d^−1^ for female), acute illness within six months of cardiovascular events, acute or chronic inflammatory or infectious diseases, or mental illness that could hinder the participants' comprehension of the nature, scope, and potential consequences of the study.

### 2.5 Detection of Intestinal Bacteria

Twenty‐four patients diagnosed with T2D or T2D with coronary calcification were included in this study. Fecal samples were collected and subjected to 16S rRNA sequencing. Fecal DNA was extracted using the Stool Genomic DNA Extraction Kit (Solarbio, Beijing, China). BF was detected using a polymerase chain reaction (PCR) assay with specific primers (Table S1, Supporting Information). The PCR reaction program consisted of an initial denaturation at 95 °C for 2 min, followed by 40 cycles of denaturation at 95 °C for 15 s and annealing at 60 °C for 30 s. The experiments were conducted using a LightCycler 480 II instrument. Furthermore, a subset of patient fecal samples underwent 16S rRNA sequencing (Genesky Bio‐Tech Co., Ltd) to identify intestinal bacteria.

### 2.6 BF Culture

BF is purchased from ATCC (25285), which is cultured in LB(Luria‐Bertani) liquid medium at 37 °C in a 5% CO_2_. A single colony of BF was selected from an LB solid plate and cultured anaerobically in LB liquid medium at 37 °C until it reached OD_600nm_ > 1.0. The bacterial solution was inoculated into 100 ml of fresh LB culture medium at a ratio of 1:50 and anaerobically cultured at 37 °C until it reached OD_600nm_ reached > 0.6. BF cells were harvested by centrifugation at 4 °C with 3000 rpm for 10 min, followed by resuspension in an appropriate volume of phosphate‐buffered saline (PBS). The quantity of BF was determined using an OD_600nm_‐bacterial number curve.

### 2.7 BF Translation

The mice were administered an antibiotic cocktail consisting of neomycin (1 mg ml^−1^), streptomycin (1 mg ml^−1^), and bacitracin (1 mg ml^−1^) in their drinking water for 3 days. Subsequently, each mouse received an intragastric administration of 0.1 ml bacterial suspension containing 5 × 10^8^ CFU ml^−1^. Mince in the control group received an intragastric administration of an equal volume of heat‐killed BF (95 °C for 30 min). This regimen was repeated twice per week for a total of four weeks.

### 2.8 Extraction of BF EV

EV was isolated using the Total Exosome Isolation Reagent Kit (Thermo Fisher Scientific, Waltham, MA, USA). Briefly, bacteria were cultured in LB liquid medium for 24 h, followed by centrifugation at 3000 g for 5 min to concentrate the bacteria. The supernatant was collected and further concentrated at 2000 g for 30 min. Subsequently, the supernatant was incubated with half the volume of EV isolation reagent at 4 °C overnight. The mixture was then centrifuged at 10000 g for 1 h, and the precipitate was resuspended in PBS. EVs were characterized using nanoparticle tracking analysis (NTA) and transmission electron microscopy, or stored at −80 °C.

### 2.9 Reconstruction of BF EV

1U DnaseI (MedChemExpress, NJ, USA) was combined with 2 µl of Lipofectamine CRISPRMAX Transfection Reagent (Invitrogen, Waltham, MA, USA) and incubated at room temperature for 20 min. Subsequently, 100 µl (1 × 10^9^) BF EVs were introduced and the mixture was incubated at 37 °C for 5 min. 50 µl volume of the exosome reagent was added for exosome extraction The exosomes were then analyzed using NTA and transmission electron microscopy, or stored at −80 °C.

### 2.10 Clearance of DNA in BF EV

As described by Yokoi A et al.^[^ ^30^ ^]^ 1U of DnaseI (MedChemExpress, NJ, USA) was incubated with 100 µl (1 × 10^9^) BF EVs at 37 °C for 30 min. Subsequently, 50 µl exosome reagent was added to extract the exosomes. The exosomes were then stored at −80 °C.

### 2.11 BF EV Experiment

In in vitro experiment: The VSMCs or RAW264.7 cells or macrophage induced by THP‐1 cells were treated with 0.5 µg ml^−1^ of BF EVs and incubated at 37 °C with 5.0% CO_2_ for 48 h. In in vivo experiment: Mice with T2D were oral gavage with 15 µg/100 µl of BF EV twice a week for four weeks.

### 2.12 IL‐4/13 Induced M2 Macrophage

As described by Lundahl MLE et al.^[^ ^31^ ^]^ the RAW264.7 cells were stimulated with 20 ng ml^−1^ of IL‐4 (MedChemExpress, NJ, USA) and 40 ng ml^−1^ of IL‐13 (MedChemExpress, NJ, USA) for 48 h at 37 °C with 5.0% CO_2_.

### 2.13 Generation of T2D Mice

Eight‐to‐ten‐week‐old C57/BL6 background mice weighing 25–30 g were obtained from the Hunan SJA Lab Animal Center in Changsha, Hunan, China. The mice were group‐housed in cages with 2–5 mice each, maintained at a controlled temperature of 22 ± 2 °C under a 12‐h light/dark cycle, and provided with ad libitum access to food and water. The mice were acclimatized to the conditions for a week before the behavioral tests were conducted. Each animal underwent only one experimental procedure. The mice were fed either a chow‐fat diet (CFD) or a high‐fat diet (HFD) (Trophic Animal Feed High‐tech Co. Ltd., Jiangsu, China) containing 10% (TP23102) or 45% (TP 23100) energy from fat, respectively. Following five weeks on the respective diets, an intraperitoneal insulin tolerance test (IPITT) was conducted, with blood glucose levels measured at 0, 15, 30, 60, and 120 min after insulin injection (Sigma Chemical, USA). Subsequently, 25 mg kg^−1^ streptozotocin (Sigma Chemical, USA) was administered intraperitoneally administered for three consecutive days. Blood glucose levels were monitored weekly via tail bleeds using a glucometer (Sannuo Biosensing Co., Ltd., Hunan, China). HFD‐fed mice exhibiting blood glucose levels ≥11.1 mm or significantly higher than CFD‐fed mice were classified as having T2D. Serum insulin levels were determined using a Mouse INS ELISA Kit (Feiya Biotechnology Co., Ltd., China). The homeostasis model assessment‐insulin resistance (HOMA‐IR) index was the following formula: HOMA‐IR = (Fasting blood glucose [mg/dL]) × (fasting serum insulin [mU/mL]) / 405.

### 2.14 Exaction of Serpine1 Protein

The mouse Serpine1 sequence, containing HRV‐3C restriction sites and histone tags at the 5′‐end coding regions, was inserted into the pcDNA3.1 plasmid. The recombinant plasmid was transfected into 293T cells and cultured for 48 h at 37 °C with 5.0% CO_2_. The Serpine1 protein was then extracted, purified using magnetic beads, subjected to HRV‐3C enzyme digestion, and the protein concentration was determined. Finally, the protein was collected and stored at −80 °C.

### 2.15 Serpine1 Experiment

In the in vitro experiment: Serpine1 (2.5ug ml^−1^) was added to the culture medium of the VSMC or RAW264.7 cells. The cells were cultured for 48 h at 37 °C with 5.0% CO2. In in vivo experiment: As described by Genestine M et al.^[^ ^32^ ^]^ T2D mice were injected with Serpine1 (4 µg/100 µl per kg bodyweight) via the tail vein twice a week for four weeks.

### 2.16 Flow Cytometry

Peripheral blood cells or RAW264.7 cells were divided into three groups (50 µl sample^−1^): blank group, isotype control group, and experimental group. The isotype control group was treated with control antibodies, while the experimental group was treated with mouse (CD11b, F4/80, CD86, and CD206) or human (CD45, CD11b, CD68, and CD206) antibodies. Samples from all the groups were incubated at room temperature for 30 min. For peripheral blood samples, an additional treatment step was included, which involved incubation with 1 ml of red blood cell lysate (BD Biosciences) for 10 min. Subsequently, the samples were resuspended in 1 ml of PBS, centrifuged at 1000 rpm for 5 min, and the resulting pellet was resuspended in 300 µl PBS before being analyzed using BD FACS AriaTM II. The details of the antibodies used are given in Supplementary Table S2 (Supporting Information).

### 2.17 Histological Analyses

The mice were deeply anesthetized using an intraperitoneal injection of 10% chloral hydrate (3.5 ml/kg). The aorta was removed and fixed in 4% paraformaldehyde. Samples of fixed alveolar lavage fluid were embedded in paraffin, and 5‐µm sections were obtained and stained with hematoxylin and eosin (HE), Alizarin red, and von Kossa stains. The samples were observed under an EVOS M7000 microscope (Invitrogen, Carlsbad, CA, USA). All detection procedures were performed in accordance with the instructions provided with the kits (Solarbio Science and Technology Co. Ltd., Beijing, China).

### 2.18 Immunohistochemistry Analysis

De‐paraffinated sections of the aorta or bone were prepared by heating the sample for 20 min, allowing it to cool at room temperature, and washing it thrice with 0.01 M PBS (pH 7.2‐7.6) for 3 min each. Endogenous peroxidase activity was blocked by incubating the sections in 1.0% periodic acid for 10 min, followed by three washes with 0.01 M PBS for 3 min each. The sections were then incubated with rabbit anti‐mouse Runx2 at 4 °C overnight. Subsequently, the sections were washed thrice with PBS for 5 min each, incubated with a secondary antibody at 37 °C for 30 min, washed thrice with PBS for 5 min each, and treated with Metal Enhanced DAB Substrate Kit (Solarbio Science and Technology Co. Ltd., Beijing, China) at room temperature for 1–5 min. After hematoxylin staining, dehydration using alcoholic, xylene treatment, and the application of a neutral resin sealing sheet, the sections were visualized using a DAB kit (ZSGB‐BIO). The details of the antibodies used are provided in Supplementary Table S2 (Supporting Information).

### 2.19 Multiplexed Immunofluorescence Staining

Immunofluorescence staining was performed using a three‐label, four‐color, multi‐fluorescence staining kit (AiFang Biological, Hunan, China). The results were analyzed using a multispectral imaging system and inFormmTM image analysis software (PerkinElmer). Following antigen retrieval, the deparaffinized sections were incubated with primary antibodies, followed by underwent secondary antibody incubation and tyramide signal amplification. Nuclei were counterstained with an anti‐fluorescence quencher containing 4′,6‐diamidino‐2‐phenylindole (DAPI). Multispectral images were captured using an Akoya Biosciences PhenolmagerTM HT. The details of the antibodies used are provided in Supplementary Table S2 (Supporting Information).

### 2.20 Index Detection of Serum

After inducing deep anesthesia in the mice, blood was obtained from the enucleated eyeball and incubated at 37 °C for 10 min. The samples were then centrifuged at 4000 rpm for 10 min, and the serum was collected for analysis using a Roche Cobas 8000 automatic biochemical analyzer.

### 2.21 Small Animal In Vivo Imaging

Three T2D mice were selected for in vivo imaging after clearing the intestinal bacteria. They were administered 100 µl of Dil‐labeled BF EVs (5 × 10^9^) once daily for two days. On the third day, small animal in vivo imaging was performed to analyze the distribution of fluorescence in the body and measure the fluorescence intensity in the tibia and femur *ex vivo*.

### 2.22 Co‐Immunoprecipitation

RAW264.7 cells were stimulated with BF EV for 48 h. After removing non‐specific protein combinations, 1 ml of macrophage protein extract was incubated with 100 µl of Protein A/G Plus Agarose (Santa Cruz Biotechnology, Inc., CA, USA), which had been washed twice and resuspended in PBS at a 50% concentration. Following protein concentration detection, 2 µg of Sting antibody was added to 500 µg of protein extract, and the mixture was gently mixed by hand and incubated at 4 °C overnight. On the next day, 50 µl of agarose was added at a 50% concentration and incubated at 4 °C for 2 h, followed by centrifugation at 2000 rpm for 5 s. Finally, Mef2d expression in the samples was detected by Western blotting. The details of the antibodies used are provided in Supplementary Table S2 (Supporting Information).

### 2.23 Transfection

After culturing the cells for 24 h until they reached a density of ≈70%, a premixed transfection mixture was added to the culture medium. This mixture consisted of 100 µl of DMEM, 2 µl of lipofectamine 3000, and either 12 pmol of siRNA or 1 µg of recombinant plasmid, which had been pre‐incubated at room temperature for 20 min. The treated cells were then cultured at 37 °C with 5.0% CO_2_ for either 24 or 48 h.

### 2.24 RNA Extraction and Complementary DNA (cDNA) Synthesis

Cellular RNA was extracted using the RNA Simple Total RNA Kit (TianGen Biotech Co., Ltd., Beijing, China). miRNA was extracted using the Mir‐XTM miRNA First Strand Synthesis Kit (Solarbio Science & Technology Co., Ltd., Beijing, China). Subsequently, cDNA was synthesized using the Revert Aid First Strand cDNA Synthesis Kit (Thermo Fisher Scientific Inc., based in Waltham, MA, USA).

### 2.25 Quantitative Reverse Transcriptase‐PCR (qRT‐PCR)

The qRT‐PCR reaction volume of 20 µl included 10 µl of 2 × SYBR Green PCR Mastermix (Takara Biomedical Technology Co., Ltd., Beijing, China), 1 µl of the forward primer, 1 µl of the reverse primer (Table S1, Supporting Information), 0.2 µl of ROX II (Beijing Solarbio Science and Technology Co., Ltd., Beijing, China), 1 µl of the cDNA template, and 6.8 µl of ddH2O. The reaction program consisted of an initial denaturation step at 95 °C for 2 min, followed by 40 cycles of denaturation at 95 °C for 15 s and annealing/extension at 60 °C for 30 s. The experiments were conducted using a LightCycler 480 II instrument.

### 2.26 Cell Protein Extraction

Cell protein was extracted using Cell Lysis Buffer for Western and IP Kit (Beyotime Biotechnology, Shanghai, China). Protein concentration was determined using a BCA Protein Assay Kit (Solarbio Science and Technology Co. Ltd., Beijing, China).

### 2.27 Western Blotting

Following sodium dodecyl sulfate polyacrylamide gel electrophoresis (SDS‐PAGE), proteins were transferred onto polyvinylidene fluoride (PVDF) membranes using a semi‐dry transfer apparatus (Bio‐Rad Laboratories, Inc., USA). Subsequently, the membrane was blocked at room temperature for 1 h with a blocking buffer consisting of Tris‐buffered saline (Applygen Technologies Inc., China), 5% Non‐fat powdered milk (Sangon Biotech Co., Ltd., Shanghai, China), and 0.1% Tween‐20 (Solarbio Science & Technology Co., Ltd., Beijing, China). The membrane was then incubated with the primary antibody overnight at 4 °C and washed thrice for 5 min each. The membrane was then exposed to a horseradish peroxidase‐linked secondary antibody at room temperature for 40 min and washed twice for 20 min each. Finally, the membrane was developed using an Immobilon Western Chemiluminescent HRP Substrate kit (EMD Millipore Corporation Burlington, MA, USA) and analyzed using a ChemiDoc TM XRS+ instrument (Bio‐Rad). the detail of antibodies used are provided in Supplementary Table S2 (Supporting Information).

### 2.28 Immunofluorescence

RAW264.7 cells were cultured on high‐adhesion glass slides and subjected to a series of treatments. The cells were fixed with 4% paraformaldehyde for 15 min, permeabilized with 0.5% Triton for 10 min, and blocked with 10% BSA for 30 min. The cells were then incubated with primary antibodies overnight, followed by incubation with secondary antibodies conjugated to Alexa Fluor‐488 (Absin Bioscience Inc., China). Finally, the cells were stained with DAPI (MedChemExpress, NJ, USA). After each step, the cells were washed thrice with PBS for 5 min each. Finally, fluorescence images were captured using an EVOS M7000 microscope (Invitrogen, Carlsbad, CA, USA). The details of the antibodies used are provided in Supplementary Table S2 (Supporting Information).

### 2.29 Chip‐Seq

The target genes of Mef2d were analyzed using the Hyperactive Universal CUT&Tag Assay Kit (Vazyme Biotech Co. Ltd., Nanjing, China). Briefly, RAW264.7 cells were collected after 48 h of BF EV stimulation. DNA sequences bound to Mef2d were collected, fragmented, purified, amplified for library preparation, and subjected to quality assessment. The DNA library was then sent to Genesky Bio‐Tech Co, Ltd., Shanghai for Illumina high‐throughput sequencing.

### 2.30 Dual‐Luciferase Assay

Wild‐type (WT) and mutant sequences of the Mef2d binding site in Trib1 and Serpine1 were chemically synthesized and cloned into the pSicheck‐2 luciferase reporter plasmid (Sangon Biotech Co. Ltd., Shanghai, China). The constructs were named Trib1‐WT, Trib1‐Mut (mutant), Serpine1‐WT, and Serpine1‐Mut (mutant). Subsequently, 500 ng of the WT or Mut plasmid and 500 ng of the pcDNA3.1 or pcDNA3.1‐Mef2d plasmid were mixed with Lipofectamine 3000 and transfected into 293T cells. The cells were then cultured at 37 °C and 5% CO_2_ for 48 h. Finally, the cells were harvested and a luciferase assay was conducted using the Dual‐Luciferase Reporter Assay System Kit (Promega, San Luis Obispo, CA, USA) at Turner BioSystems (Sunnyvale, CA, USA).

### 2.31 Enzyme‐Linked Immunosorbent Assay (ELISA)

The concentrations of Serpine1 in the culture medium of RAW264.7 or THP‐1 cells, as well as that in the serum of mice, patients with T2D, and patients with T2D VC, were measured using ELISA kits designed for mice or humans (Jiangsu Meimian Industrial Co, Ltd, China).

### 2.32 Macrophage Clearance in T2D Mice

Macrophages clearance in T2D mice was conducted using chodronate liposomes and control liposomes kits (Liposoma, Target Technology Co, Ltd., Beijing, China). Specifically, 10 T2D mice were divided into two groups: one group received an intravenous injection of 0.1 ml of chodronate liposomes per mouse, while the other group received an intravenous injection of 0.1 ml of control liposomes per mouse, once every week for 7 weeks. After 24 h, BF EV was administered to the mice at a dose of 15 µg/100 µl, twice a week for 8 weeks.

### 2.33 Bone Marrow Transplantation

As described by Yamasuji‐Maeda et al.^[^ ^33^ ^]^ a cell suspension of 5 × 10^7^ cells in PBS were prepared and stored at 4 °C prior to transplantation. WT mice were anesthetized with isoflurane, the hair was shaved from the groin to the knee joint area, and a 26‐gauge needle was used to enter the tibial cavity from the patellar tendon. Each mouse received an injection of 10 µl of bone marrow cells derived from T2D mice, while the control group was injected with an equal volume of PBS.

### 2.34 Transcriptome Sequencing

RAW264.7 cells were treated with BF EV at 37 °C and 5.0% CO2 for 48 h. The cells were then collected and analyzed using transcriptome sequencing (RiboBio Guangzhou, China).

### 2.35 Bioinformatic Analysis

Cluster heatmap and Venn diagram analyses were conducted using http://www.bioinformatics.com.cn/. Homology modeling was performed using https://swissmodel.expasy.org/interactive. The interactions between Mef2d and Sting was investigated using rigid protein‐protein docking on https://gramm.compbio.ku.edu/gramm. The docking sites were identified using the module available at https://www.ebi.ac.uk/pdbe/pisa/. The Molecular 3D viewer available at https://www.rcsb.org/3d‐view. Gene ontology (GO) analysis of the cell membrane proteins was conducted using https://geneontology.org/. Gene expression omnibus (GEO) data were downloaded from https://www.ncbi.nlm.nih.gov/gds/. National Center for Biotechnology Information (https://www.ncbi.nlm.nih.gov/) was used to search for protein and nucleic acid sequences. Primers were designed using the Lasergene software.

### 2.36 Statistical Analysis

All experiments were conducted with a minimum of 3 repetitions. Data analysis was carried out using either GraphPad Prism 8.00 or IBM SPSS Statistics 22 softwares. The results are presented as mean ± standard error of the mean (s.e.m). Statistical significance was assessed using Student's t‐test or one‐way analysis of variance (ANOVA) with Bonferroni correction. A multiparameter logistic regression analysis of the clinical data was conducted Results with *p* < 0.05 were considered statistically significant, while results with *p* < 0.01 were considered very significant.

## 3 Results

### 3.1 BF is a T2D VC‐Related Intestinal Bacterium Associated with Macrophage M2 Polarization

**Figure 1:**
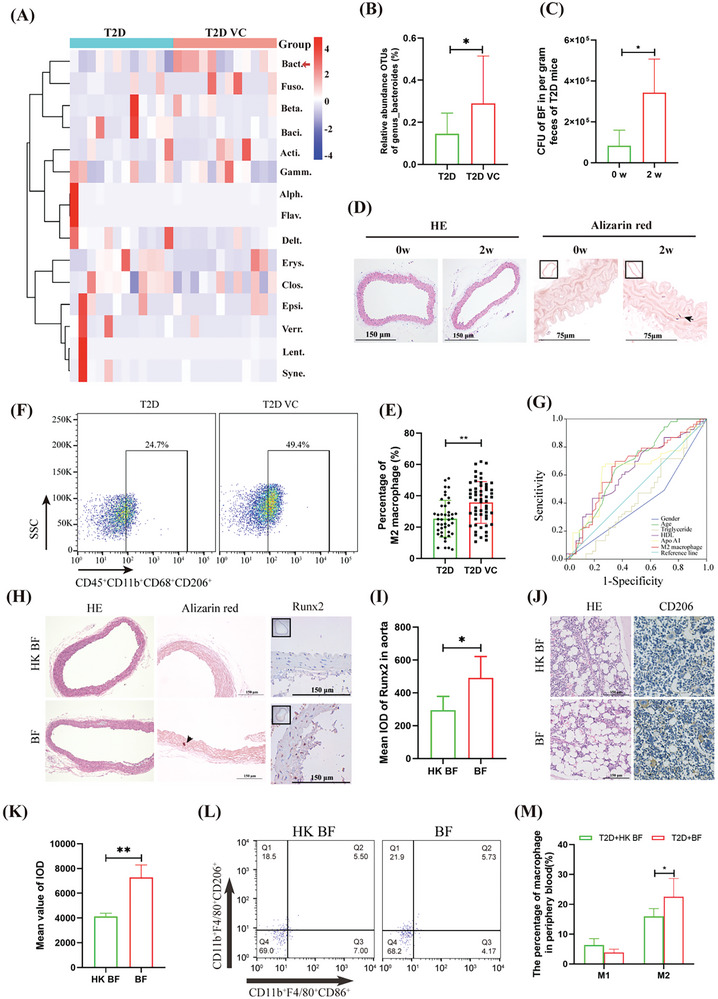
Analysis of intestinal bacteria and action pathways related to T2D VC. A)16s rRNA sequencing analysis of intestinal bacterial abundance in patients with T2D and T2D VC. B) Analysis of BF levels in patients with T2D and T2D VC. C) qRT‐PCR analyzed the impact of the progression of T2D on intestine BF. D) Pathological analyzed the effects of the progression of T2D on aortic calcification. E,F) Flow cytometry analysis of expression changes of peripheral M2 monocytes/macrophages in T2D and T2D VC patients (E), and statistical analysis (F). G) ROC analysis of AUC of significant factors related to T2D VC. H,I) Pathological and immunohistochemical analyzed the effect of BF transplantation on the aorta of T2D mice (H), and statistical analysis (I). J,K) Pathological and immunohistochemical analyzed the effect of BF transplantation on bone marrow cells in T2D mice (J), and statistical analysis (K). L,M) Flow cytometric analyzed the effect of BF transplantation on peripheral monocytes/macrophage polarization (L), and statistical analysis (M). Animal experiments included ≧5 mice, the experiment was repeated three times, the results are expressed as mean ± standard error, ^*^ means *p* < 0.05, ^**^means *p* < 0.01.

This study collected fecal samples from 12 patients with T2Dand T2D VC. We used 16S rRNA sequencing analysis and found that only Bacteroidetes expression was significantly up‐egulated (**Figure** **1**A), with BF exhibiting a notable 2.00‐fold increase (Figure 1B). Analysis of the T2D mouse model revealed that intestinal BF levels were significantly higher two weeks after the onset of T2D (Supplementary Figure S1A—D, Supporting Information), showing a 2.91‐fold increase compared to baseline levels (0 weeks) (Figure 1C). Further, analysis of the peripheral blood and bone marrow revealed significant polarization of macrophages toward the M2 phenotype (Supplementary Figure S1E,F, Supporting Information). Calcium ion deposition was also observed in the aorta (Figure 1D). In comparison, patients with T2D VC showed a significant 1.41 fold increase in peripheral M2 polarization of monocytes/macrophages (25.3% vs 35.72%) (Figure 1E,F). Among the six indicators that differed significantly between patients with T2D and those with T2D VC (gender, age, triglycerides, high density lipoprotein (HDL), apolipoprotein A1, and M2 macrophages) (Table S3, Supporting Information), the area under the receiver operating characteristic (ROC) curve for M2 macrophages was the largest for the serological indicators (area under the curve [AUC] = 0.646, p < 0.05), with a sensitivity of 69.8% and specificity of 65.1% (Figure 1G; **Table** **1**). Furthermore, BF cultured in vitro was transplanted into the intestinal tracts of T2D mice. The results demonstrated significant 1.77‐fold and 1.67‐fold increases in the expression levels of CD206 in the bone marrow and Runx2 in the aorta, respectively, and accompanied by calcium ion deposition (Figure 1H–K). Flow cytometry analysis revealed that the M2 polarization of monocytes/macrophages in the peripheral blood increased significantly (1.41‐fold), while no significant difference was observed in the M1 polarization of monocytes/macrophages (Figure 1L,M). These findings suggest that BF is an intestinal bacterium associated with T2D VC and is related to M2 macrophage levels.

**Table 1:** Comparative analysis of predictive factors in T2D VC patients.

Test variable	AUC	95% CI of AUC		Sensitivity	Specificity	Standard Error	*p*
		lower bound	upper bound				
Gender	0.396	0.283	0.510	0.491	0.302	0.058	0.082
Age	0.663	0.551	0.775	0.642	0.651	0.057	0.006^*^
Triglycerides	0.430	0.312	0.747	0.925	0.140	0.060	0.237
HDL	0.626	0.513	0.739	0.434	0.814	0.058	0.034^*^
Apolipoprotein A1	0.624	0.509	0.739	0.660	0.744	0.059	0.037^*^
M2 macrophage	0.646	0.533	0.760	0.698	0.651	0.058	0.014^*^

Note: AUC represents the area under the curve; CI is the confidence interval. Significance difference is labeled with one star (*p* < 0.05).

### 3.2 BF Aggravates Osteogenic Differentiation of VSMC by Inducing Macrophages M2 Polarization in the Bone Marrow

The causal relationship between intestinal BF and the activation of bone marrow macrophages was clarified by transplanting bone marrow from T2D mice into wild‐type (WT) mice. The results demonstrated significant polarization of monocytes/macrophages toward the M2 phenotype in the peripheral blood (Supplementary Figure S1G,H, Supporting Information). Furthermore, the expression levels of the miR‐32, Runx2, and Alp genes in the aorta were significantly up‐egulated 2.07‐, 1.73‐, and 1.39‐folds, respectively (Supplementary Figure S1I, Supporting Information). However, no significant change was observed in the BF levels in the mouse intestine (Supplementary Figure S1J, Supporting Information). These findings suggest that pathological changes in bone marrow cells are crucial factors contributing to VC in T2D mice.

### 3.3 EV Production is an Important Way for BF to Induce M2 Macrophages and Promote T2D VC

**Figure 2:**
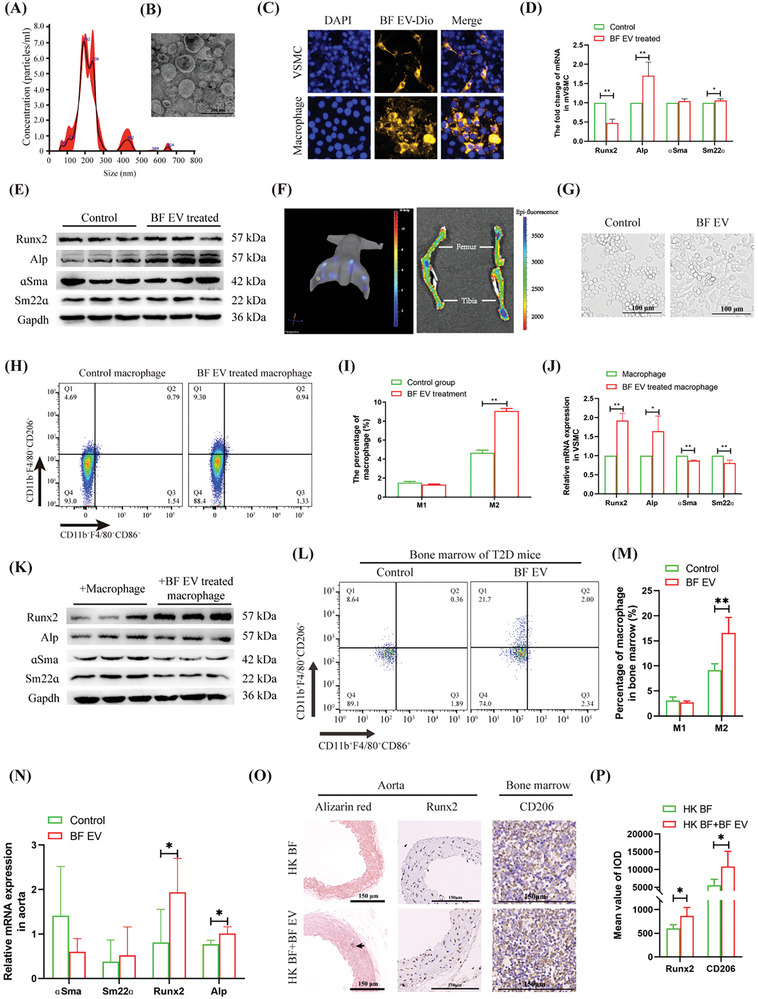
Analyzed the effects of BF EV on VC and macrophage polarization. A) NTA analysis of vesicle size distribution of BF. B) Scanning electron microscopy analysis of BF EV structure. C) Observation of macrophages and VSMCs up‐taken BF EV. D) qRT‐PCR analyzed the effect of BF EV on VSMC osteogenic marker genes. E) Western blotting analyzed the effect of BF EV on VSMC osteogenic marker genes. F) Small animal in vivo imaging analyzed the distribution of BF EV in vivo. G) Light microscopy analyzed the effect of BF EV on macrophage morphology. H,I) Flow cytometric analyzed the effect of BF EV on macrophage polarization (H), and statistical analysis (I). J) qRT‐PCR analyzed the effect of BF EV‐activated macrophages on VSMC osteogenic marker genes. K) Western blotting analysis of the effect of BF EV‐activated macrophages on VSMC osteogenic marker genes. L,M) Flow cytometry analyzed the effect of BF EV on bone marrow macrophage polarization in T2D mice (L), and statistical analysis (M). N) qRT‐PCR analyzed the effect of BF EV on aortic calcification in T2D mice. O,P) Pathological and immunohistochemical analyzed BF EV antagonizing the inhibitory effect of HK BF on aortic calcification and bone marrow M2 macrophages in T2D mice (O), and statistical analysis (P). Animal experiments included ≧5 mice, the experiment was repeated three times, the results are expressed as mean ± standard error, ^*^ means *p* < 0.05, ^**^means *p* < 0.01.

EV serve as crucial carriers of information exchange across tissues, cells, or even species. In this study, BF EV was extracted using a specialized kit, yielding an average particle size of 223.3 ± 21.9 nm (**Figure** **2**A). These EV exhibit a double‐membrane, cup‐shaped structure (Figure 2B) and can be internalized by VSMCs and macrophages (Figure 2C). The Alp gene was significantly upregulated when the VSMCs were stimulated with BF EV; however, Runx2 was notably downregulated, and smooth muscle markers did not show significant changes (Figure 2D,E). In vivo small animal imaging revealed that BF EV accumulated in the tibia and abdominal cavity of mice after they were oral gavage (Figure 2F). When macrophages were stimulated in vitro with BF EV, they adopted a spindle shape characteristic of M2 macrophages (Figure 2G); flow cytometry confirmed the significant polarization of macrophages toward the M2 phenotype (Figure 2H,I). Interestingly, co‐culturing VSMCs with macrophages stimulated by BF EV resulted in a significant upregulation of Runx2 and Alp, and a significant downregulation of ɑSma and Sm22ɑ (Figure 2J,K). At the animal level, administration of BF EV to T2D mice led to a significant 1.81 fold increase (from 9.14% to 16.58%) in M2 macrophages in the bone marrow to (Figure 2L,M), along with a significant upregulation of Runx2 and Alp in the mouse aorta (Figure 2N). Furthermore, after blocking the colonization site of BF in the intestines of T2D mice with (heat killed) HK BF, intragastric administration of BF significantly promoted aortic osteogenic differentiation and M2 polarization of bone marrow macrophages (Figure 2O,P; Figure S3A,B, Supporting Information). These results suggest that BF induces macrophage M2 polarization via EV, thereby promoting aortic calcification.

### 3.4 Clearing Macrophages in T2D Mice Alleviates BF EV‐Induced VC

**Figure 3:**
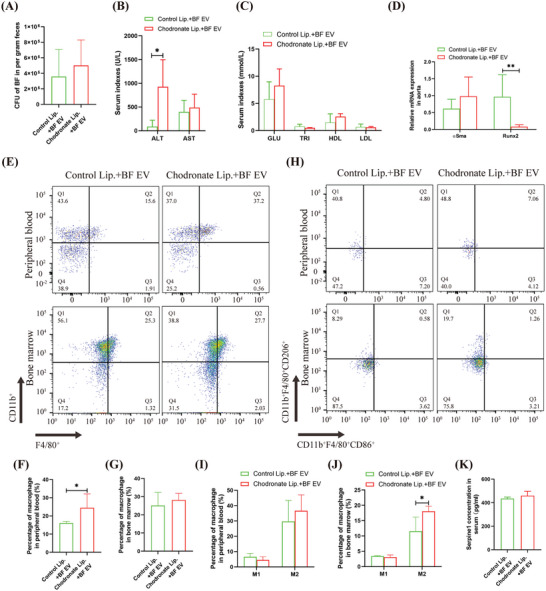
Analyzed the effect of macrophage clearance on BF EV exacerbated T2D VC in mice. A)qRT‐PCR detection of fecal BF. B) Serum index of liver function detection. C) Serum index of blood glucose and blood lipid detection. D) qRT‐PCR detected the expression of aortic osteogenic differentiation marker gene. E–G) Flow cytometry analysis of total macrophages in peripheral blood and bone marrow (E), and statistical analysis (F, G). H–J) Flow cytometry analysis of M1/M2 macrophages in peripheral blood and bone marrow (H), and statistical analysis (I, J). K) ELISA detected the level of Serpine1 in Serum. Animal experiments included ≧4 mice, the experiment was repeated three times, the results are expressed as mean ± standard error, ^*^ means *p* < 0.05, ^**^means *p* < 0.01.

After mice were administrated with chodronate liposomes, results showed effectively clear macrophages from the peripheral blood, bone marrow, and spleen of the mice within 24 h (Supplementary Figure S5, Supporting Information). Consequently, T2D mice were infused with chodronate liposomes via the tail vein and intragastrically administered BF EV (**Figure** **3**). We found no significant change in the number of fecal BF between the chodronate liposomes group compared to the control liposome group (Figure 3A). Additionally, there were no significant changes in aspartate aminotransferase (AST), blood glucose, or blood lipids (triglycerides, HDL, and low density lipoprotein (LDL)); however, alanine aminotransferase (ALT) levels increased significantly (10.44 fold; 88.75 U L^−1^ vs 926.2 U L^−1^; Figure 3B,C). Especially importantly, qRT‐PCR analysis demonstrated that Runx2 was significantly downregulated by 12.08‐fold (0.96 vs 0.08) in the aorta (Figure 3D). Flow cytometry revealed that the total macrophage count in the peripheral blood increased significantly (1.52 fold; 16.05% vs 24.44%); there were no significant change in the M1/M2 monocyte/macrophage ratio in the peripheral blood (Figure 3E–G). While there was no significant change in the numbers of M1 macrophages in the bone marrow, the numbers of M2 macrophages increased significantly (1.57 fold; 11.53% vs 18.06%; Figure 3H–J). ELISA indicated that after macrophage clearance, BF EV transplantation had no significant effect on serum Serpine1 levels in T2D mice (433.9 pg ml^−1^ vs 459.0 pg ml^−1^) (Figure 3K). These results suggest that macrophages are the key target cells for the promotion of T2D VC by BF EV.

### 3.5 BF EVs Promote Macrophage M2 Polarization Through Sting‐Mef2d Signaling

**Figure 4:**
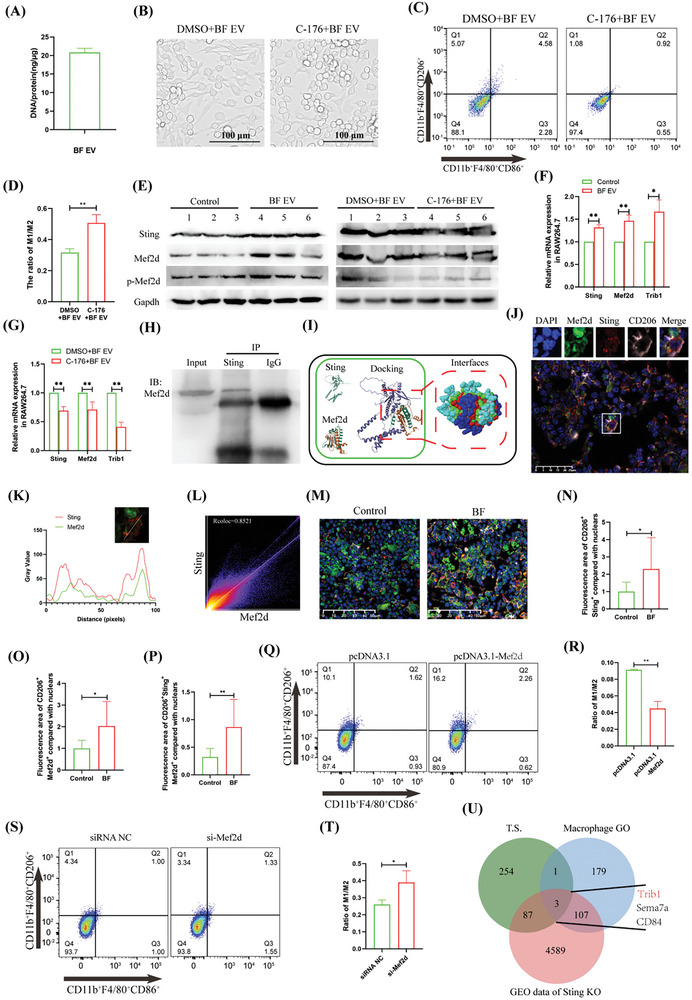
BF EV activated Sting‐Mef2d signaling to promote macrophage M2 polarization. A) Detection of DNA content in BF EV. B) Optical microscopy observation that inhibiting Sting activity antagonized BF EV‐induced macrophage M2 polarization. C,D) Flow cytometric analyzed inhibition of Sting activity antagonizing BF EV‐induced macrophage M2 polarization (C), and statistical analysis (D). E) Western blotting analysis of the effects of BF EV and C‐176 + BF EV treatment on macrophage gene expression. F) qRT‐PCR analyzed the effect of BF EV on macrophage gene expression. G) qRT‐PCR analyzed the effect of C‐176 + BF EV treatment on macrophage gene expression. H) Co‐IP analysis of the interaction between Sting and Mef2d. I) Bioinformatics analysis of Sting and Mef2d's Docking. J–L) Tissue immunofluorescence analyzed the effect of BF transplantation on bone marrow gene distribution in T2D mice (J), colocalization curve of Sting and Mef2d (K), and colocalization scatter plot analysis (L). M–P) Tissue immunofluorescence analyzed the effect of BF transplantation on bone marrow gene expression in T2D mice (,), and statistical analysis of CD206^+^Sting^+^ (N), CD206^+^Mef2d^+^ (O) and CD206^+^Sting^+^Mef2d^+^ (P). Q,R) Flow cytometric analyzed the effect of Mef2d overexpression on macrophage polarization (Q), and statistical analysis (R). S,T) Flow cytometric analyzed the effect of Mef2d inhibited expression on macrophage polarization (S), and statistical analysis (T). U) Transcriptome sequencing and bioinformatics analysis of genes regulated by Mef2d. The experiment was repeated three times, the results are expressed as mean ± standard error, ^*^ means *p* < 0.05, ^**^means *p* < 0.01.

EVs induce immune cell activation through Sting signaling, which is mediated by the nucleic acids they carry. We found that 7 × 10^8^ particles ml^−1^ of BF EV contained 20.84 ng µl^−1^ of dsDNA (**Figure** **4**A). Light microscopy observations and flow cytometry analyses revealed that the inhibition of Sting activation in macrophages using C‐176 effectively antagonized the BF EV‐induced polarization of macrophages into an elongated spindle‐shaped M2 phenotype (Figure 4B–D). When the BF EVs were treated directly with vesicle recombination agents or DNaseI, EV recombination did not remove DNA from the BF EVs effectively (Supplementary Figure S3C–F, Supporting Information); however, treatment with DNaseI significantly reduced the DNA concentration (1.6 fold; 2 to 1.25 ng µl^−1^; Figure S3F, Supporting Information). While reducing the DNA content of EV had no significant effect on macrophage proliferation (Supplementary Figure S3G, Supporting Information), it significantly reduced the proportion of M2 macrophages (1.56‐fold; Supplementary Figure S3H,I, Supporting Information) and downregulated the Sting pathway (Supplementary Figure S3J,K, Supporting Information). Mef2d is a crucial gene associated with M2 polarization of macrophages and has been extensively studied. qRT‐PCR and Western blot analyses indicated that during BF EV‐induced macrophage M2 polarization, the Mef2d and phosphorylated Mef2d levels changed in the same manner as the changes in Sting levels (Figure 4E–G). Co‐IP confirmed that Mef2d binds to Sting (Figure 4H). Bioinformatics analysis further revealed that Mef2d and Sting surface molecules could form better chimeras, with hydrogen bonds observed between specific amino acids (Figure 4I; **Table** **2**). Notably, reducing the DNA content of vesicles significantly downregulated Mef2d expression (Supplementary Figure S3J,K). Multicolor tissue immunofluorescence analysis revealed that Sting and Mef2d co‐localized in the tibia of BF‐transplanted T2D mice (Figure 4J–L). The signals for CD206^+^Sting^+^, CD206^+^Mef2d^+^, and CD206^+^Sting^+^Mef2d^+^ were significantly elevated compared with those in the control groups, with increases of 2.30‐, 2.29‐, and 2.68‐fold increases, respectively (Figure 4M–P). Furthermore, flow cytometry and immunofluorescence analysis indicated that Mef2d overexpression significantly induced a 2.03‐fold increase in M2 macrophages (Figure 4Q,R), whereas Sting levels showed no significant change (Supplementary Figure S2A,C,D). In contrast, Mef2d inhibiting resulted in a significant 1.50‐fold increase in M1 macrophage polarization, reaching (Figure 4S,T), while Sting levels remained unchanged (Figure S2A,B,E, Supporting Information). Moreover, qRT‐PCR and Western blotting performed after inducing M2 macrophages polarization with IL‐4/13 (Supplementary Figure S2H,I, Supporting Information) demonstrated that while Sting levels did not exhibit significant changes, Mef2d was significantly up‐regulated (Supplementary Figure S2F,G, Supporting Information). These findings suggest that Sting is a key gene in BF EV‐induced macrophage M2 polarization, and that Mef2d is as an important downstream functional gene activated by BF EV.

**Table 2:** Results of molecular docking.

Receptor	Ligand	Hydrogen Bond		Salt Bridges	
Mef2d	Sting	Interaction	Distance [Å]	Interaction	Distance [Å]
		A:ARG 24[NH1]‐ B:GLU 34[OE1]	2.55	A:ARG 24[NH1]‐ B:GLU 34[OE1]	2.55
		A:ILE 11[N]‐ B:LEU 38[O]	2.76	A:ARG 10[NE]‐ B:ASP 40[OD1]	3.40
		A:TYR 72[OH]‐ B:ASN 52[O]	3.01	A:GLU 34[OE1]‐ B:ARG 24[NH1]	2.83
		…	…	…	…

### 3.6 Trib1 is a Key Target Gene for BF EV‐Sting‐Mef2d Signaling to Promote M2 Macrophages Polarization

**Figure 5:**
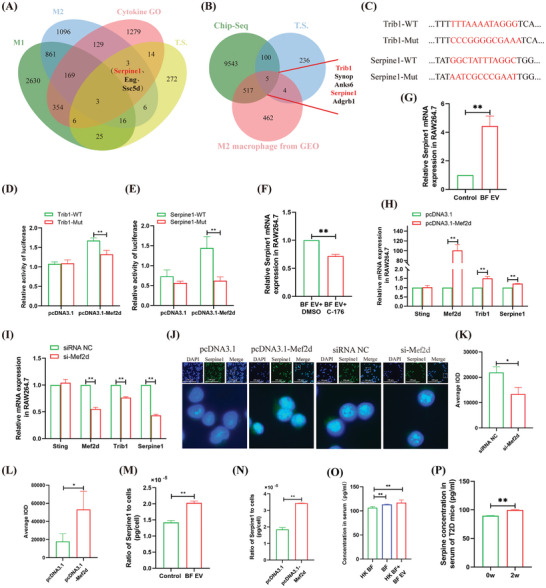
Analysis of the mechanism by which Mef2d promotes macrophage M2 polarization and cytokine secretion. A) Venn diagram analysis of BF EV‐induced cytokine secretion by M2 macrophages. M1 and M2 are the transcriptome data of human monocytes derived from GEO after inducing macrophage polarization (GSE157182). TS is BF EV processing transcriptome sequencing data of RAW264.7 cells. B) Venn diagram analyzed target genes regulated by Mef2d. Chip‐Seq is the sequencing data of Mef2d. C) Mef2d binding sites and mutation sites on the non‐coding regions of Trib1 and Serpine1. D) Dual‐luciferase assay to analyze the binding of Mef2d to the non‐coding region of Trib1. E) Dual‐luciferase assay to analyze the binding of Mef2d to the non‐coding region of Serpine1. F) qRT‐PCR analyzed the effect of BF EV on macrophage cytokine expression. G) qRT‐PCR analyzed the effect of C‐176 + BF EV on macrophage cytokine expression. H) qRT‐PCR analyzed the effect of Mef2d overexpression on gene expression. I) qRT‐PCR analyzed the effect of si‐Mef2d transfection on gene expression in macrophages. J–L) Immunofluorescence analyzed the effect of pcDNA3.1‐Mef2d or si‐Mef2d transfection on Serpine1 expression in macrophages (J), and statistical analysis (K,L). M–P) ELISA detected the level of Serpine1 in culture supernatant of BF EV treated macrophages (M) or pcDNA3.1‐Mef2d transfected macrophages (N), and in serum of BF/BF EV transplanted T2D mice (O) or T2D mice during disease progression. Animal experiments included ≧4 mice, the experiment was repeated three times, the results are expressed as mean ± standard error, ^*^ means *p* < 0.05, ^**^means *p* < 0.01.

Venn diagram analysis based on transcriptome sequencing data from BF EV‐treated macrophages, Sting knockout mouse macrophages infected with *Mycobacterium tuberculosis* (GSE162620), and macrophage GO of macrophage, showed that Trib1 was significantly associated with M2 macrophages and Sting activation (Figure 4U). Additionally, Venn diagram analysis based on Mef2d ChIP‐seq data, transcriptome sequencing of BF EV‐treated macrophages, and transcriptome sequencing of human peripheral monocytes differentiated into M2 macrophages (GSE157182), revealed that Trib1 is a target gene of Mef2d (**Figure** **5**B). Dual‐luciferase assay showed that Mef2d promoted the expression of Trib1 (Figure 5B–D). Following treatment with BF EV or C‐176 + BF EV, Trib1 expression levels in the macrophages were upregulated 1.66‐fold or downregulated 2.44‐fold, respectively (Figure 4F,G). Reduction in EV DNA significantly decreased Trib1 expression (Supplementary Figure S3J, Supporting Information). Immunofluorescence analysis demonstrated that Mef2d overexpression led to a 4.32‐fold increase in Trib1 expression, whereas Mef2d inhibition resulted in a 2.59‐fold decrease in Trib1 expression (Supplementary Figure S2A—C, Supporting Information). Furthermore, qRT‐PCR analysis performed after inducing M2 macrophages polarization with IL‐4/13 (Supplementary Figure S2H,I, Supporting Information) revealed significant upregulation of Trib1 expression (Supplementary Figure S2F, Supporting Information). Notably, Trib1 overexpression antagonized the induction of M1 macrophages polarization through si‐Mef2d (Supplementary Figure S4, Supporting Information). These findings suggest that Trib1 is a key gene through which Mef2d promotes M2 polarization of macrophages.

### 3.7 BF EV‐Sting‐Mef2d Signaling Promotes the Secretion of Serpine1 from M2 Macrophages to Exacerbate T2D VC

Venn diagram analysis of cytokine GO terms was performed based on transcriptome sequencing data from BF EV‐treated macrophage and human peripheral blood monocytes differentiated into M1 or M2 macrophages (GSE157182). The analysis identified Serpine1, Eng, and Ssc5d as common genes (Figure 5A). Furthermore, ChIP‐seq data for Mef2d identified Serpine1 as a potential target gene for Mef2d transcriptional regulation (Figure 5B). A dual‐luciferase assay confirmed that Mef2d has a binding site within the Serpine1 gene (Figure 5C,E).

qRT‐PCR analysis revealed that BF EV significantly upregulated (4.44 fold) Serpine1 expression in macrophages (Figure 5F). In contrast, treatment with C‐176 + BF EV resulted in a significant downregulation (1.39 fold) of Serpine1 (Figure 5G). A reduction in vesicle DNA content also led to a significant downregulation of Serpine1 expression (Figure S3J, Supporting Information). Furthermore, macrophages transfected with pcDNA3.1‐Mef2d exhibited a significant up‐regulation (1.22‐fold) of Serpine1 (Figure 5H), whereas macrophages transfected with si‐Mef2d showed a significant down‐regulation of Serpine1 by 2.31‐fold (Figure 5I). Immunofluorescence analysis corroborated these findings, demonstrating that macrophages transfected with pcDNA3.1‐Mef2d significantly upregulated (2.98‐fold) Serpine1, while those transfected with si‐Mef2d downregulated (1.64‐fold) Serpine1 (Figure 5J–L). ELISA indicated that BF EV significantly increased (1.43‐fold; 1.421 pg cell^−1^ vs 2.032 pg cell^−1^) the secretion of Serpine1 in macrophages (Figure 5M), and Mef2d overexpression further promoted (1.86‐fold; 1.845 pg cell^−1^ vs 3.431 pg cell^−1^) Serpine1 secretion (Figure 5N). Additionally, BF‐ and BF EV‐transplantation significantly upregulated serum Serpine1 levels in T2D mice (BF: 1.06‐fold, 106.6 pg ml^−1^ vs 113.5 pg ml^−1^); BF EV: 1.10‐fold, 106.6 pg ml^−1^ vs 117.0 pg ml^−1^; Figure 5O). Notably, Serpine1 was significantly upregulated (1.11 fold; 89.73 pg ml^−1^ vs 99.58 pg ml^−1^) in the serum of mice after 2 weeks of T2D progression (Figure 5P). These results indicate that Serpine1 is a crucial protein for Mef2d to promote the secretion of M2 macrophages.

### 3.8 Serpine1 Promotes T2D VC

**Figure 6:**
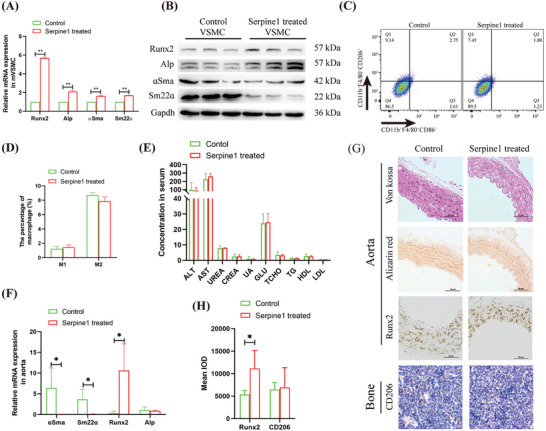
Analyzed the effect of Serpine1 on VC in T2D mice. A) qRT‐PCR analyzed the effect of Serpine1 on the expression of VSMC osteogenic differentiation marker genes. B) Western blotting analyzed the effect of Serpine1 on the expression of osteogenic differentiation marker genes in VSMC. C,D) Flow cytometry analyzed the effect of Serpine1 on macrophage polarization (C), and statistical analysis (D). E) Biochemical detected the effect of Serpine1 infusion on liver and kidney function in T2D mice. F) qRT‐PCR analyzed the effect of Serpine1 infusion on the expression of genes related to osteogenic differentiation in the aorta of T2D mice. G,H) Pathological and immunohistochemical analyzed the effect of Serpine1 infusion on aortic Runx2 and bone marrow CD206^+^ cells in T2D mice (G), and statistical analysis (H). Animal experiments included ≧4 mice, the experiment was repeated three times, the results are expressed as mean ± standard error, ^*^ means *p* < 0.05, ^**^means *p* < 0.01.

Mouse Serpine1 protein of mice was extracted using prokaryotic expression and purification techniques. qRT‐PCR performed after the stimulation of mouse VSMCs with Serpine1 revealed that the expression levels of Runx2, Alp, αSMA, and Sm22α were significantly up‐regulated by 5.67‐, 2.1‐, 1.63‐, and 1.7‐folds, respectively (**Figure** **6**A). Western blot analysis confirmed that Runx2 and Alp were significantly upregulated, while αSMA and Sm22α exhibited significant downregulation (Figure 6B). In vitro experiments indicated that Serpine1 had no significant effect on macrophage polarization (Figure 6C,D). In vivo experiments demonstrated that the infusion of Serpine1 through the tail vein of T2D mice did not result in significant changes in liver and kidney function (Figure 6E). Furthermore, qRT‐PCR analysis indicated that Runx2 expression was significantly increased (31.10‐fold) in the aorta, whereas αSMA and Sm22α were significantly down‐regulated (101.92‐fold and 49.30‐fold, respectively; Figure 6F). Pathological staining and immunohistochemical analyses revealed that Serpine1‐treated mice did not exhibit obvious calcification points; however, Runx2 levels were significantly increased (2.08 fold), and there was no significant change in the population of bone marrow CD206^+^ cells (Figure 6G,H). Together, these results suggest that Serpine1 promotes vascular osteogenic differentiation in T2D mice.

### 3.9 Peripheral Blood Serpine1 is the Risk Factor for T2D VC

**Figure 7:**
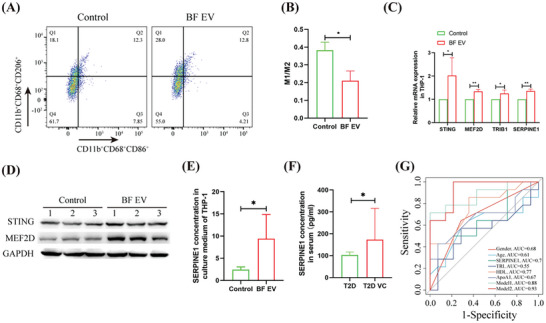
Clinical level analysis of the relationship between serum Serpine1 and T2D VC. A,B) Flow cytometric analyzed the effect of BF EV on the polarization of THP‐1‐derived macrophages (A), and statistical analysis (B). C) qRT‐PCR analyzed the effect of BF EV on gene expression of THP‐1‐derived macrophages. D) Western blotting analyzed the effect of BF EV on gene expression of THP‐1‐derived macrophages. E) ELISA analyzed the effect of BF EV on Serpine1 secretion from THP‐1‐derived macrophages. F) ELISA analysis of serum Serpine1 levels in T2D VC patients. G) Logistic regression analysis of the correlation between Serpine1 and T2D VC, model 1: gender, age, triglyceride (TRI), high‐density lipoprotein (HDL), apolipoprotein A1 (ApoA1); model 2: Gender, age, triglyceride (TRI), Serpine1, high‐density lipoprotein, apolipoprotein A1. The experiment was repeated three times, the results are expressed as mean ± standard error, ^*^ means *p* < 0.05, ^**^means *p* < 0.01.

The effect of BF EV on human macrophages and their relationship with T2D VC were elucidated by differentiating THP‐1 cells into macrophages and then treating them with BF EV. Flow cytometry analysis demonstrated significant polarization of macrophages toward the M2 phenotype, exhibiting a 1.82 fold increase (**Figure** **7**A,B). qRT‐PCR analysis revealed that Sting, Mef2d, Trib1, and Serpine1 were all significantly upregulated, with 2.02‐, 1.34‐, 1.25‐, and 1.35‐fold increases, respectively (Figure 7C). Western blotting confirmed that both Sting and Mef2d were significantly upregulated following BF EV stimulation (Figure 7D). ELISA indicated that Serpine1 was significantly elevated in the macrophage culture supernatant (3.85‐fold; 2.44 pg ml^−1^ vs 9.42 pg ml^−1^; Figure 7E) after BF EV stimulation and in T2D VC serum (1.68‐fold; 102.8 pg ml^−1^ vs 172.9 pg ml^−1^; Figure 7F). Logistic regression analysis indicated that the model constructed with Serpine1, gender, age, triglycerides, HDL, and apolipoprotein A1 yielded an AUC of 0.929 (*p* < 0.01), with a sensitivity of 100.0% and a specificity of 21.4% (Figure 7G; **Table** **3**). These findings suggest that macrophage M2 polarization and the secretion of Serpine1 are significant risk factors for T2D VC.

**Table 3:** Comparative analysis of Serpine1 and predictive factors in T2D VC patients.

Test variable	AUC	95% CI of AUC		Sensitivity	Specificity	Standard Error	*p*
		lower bound	upper bound				
Gender	0.321	0.118	0.525	0.357	0.714	0.104	0.108
Age	0.607	0.386	0.828	0.643	0.357	0.113	0.335
Serpine1	0.671	0.464	0.878	0.429	0	0.106	0.124
Triglycerides	0.449	0.226	0.672	0.214	0.071	0.114	0.646
HDL	0.765	0.588	0.943	0.857	0.357	0.091	0.017*
Apolipoprotein A1	0.671	0.463	0.878	0.429	0.071	0.106	0.124
Model 1	0.878	0.745	1	0.786	0.143	0.067	0.001*
Model 2	0.929	0.836	1	1	0.214	0.047	0*

Note: AUC represents the area under the curve; CI is the confidence interval; Model1: gender, age, triglycerides, HDL, and apolipoprotein A1; Model2: Serpine1, gender, age, triglycerides, HDL, and apolipoprotein A1. Significance difference is labeled with one star (*p* < 0.05).

## 4 Discussion

Intestinal flora and genetic, environmental, and dietary factors are closely linked to the occurrence and progression of T2D.^[^ ^34^ ^]^ Intestinal flora maintains a dynamic physiological balance that is essential for normal bodily functions;^[^ ^35^ ^]^ In a pathological state, intestinal flora often becomes disordered, which can predispose individuals to diabetes and its complications.^[^ ^36^, ^37^ ^]^ Research has shown that the oral administration of beneficial bacteria and pharmacological agents, or other strategies to modulate intestinal bacteria can significantly improve clinical outcomes in patients with diabetes.^[^ ^38^, ^39^, ^40^ ^]^ Therefore, investigating the intestinal bacteria associated with T2D VC and their mechanisms of action are crucial for the prevention and treatment of T2D.

In this study, we identified intestinal BF as a bacterium associated with vascular complications in T2D, which exacerbates these complications by inducing M2 polarization of bone marrow macrophages (Figure 1; Figure S1, Supporting Information). BF is an obligate anaerobic bacterium that is recognized as a primary commensal organism and an important pathogenic microorganism in the colons of many mammals.^[^ ^41^, ^42^, ^43^ ^]^ It is also closely assocoated with the progression of diabetes.^[^ ^44^, ^45^, ^46^ ^]^ Currently, BF has emerged as a crucial intestinal bacterium in clinical settings.^[^ ^47^, ^48^ ^]^ Consequently, elucidating the mechanisms by which BF regulates the vascular complications associated with T2D has substantial clinical significance and value.

Intestinal bacteria regulate the biological activity of target cells over long distances through the production of EV.^[^ ^49^, ^50^ ^]^ In this study, we observed that aortic osteogenic differentiation was enhanced following the oral gavage of BF EV in T2D mice (Figure 2). However, in vitro experiments revealed a significant decrease in Runx2 expression in VSMC after treatment with BF EV (Figure 2). Runx2 is a crucial initiating gene for the osteogenic differentiation of VSMC,^[^ ^51^ ^]^ and its significant upregulation is regarded as an important marker of this differentiation process.^[^ ^7^ ^]^ These findings indicate that BF EV do not directly promote T2D VC, but rather facilitate T2D VC through indirect pathways. Liu et al. found that intestinal bacterial EV influence the metabolic activity of liver macrophages via the peripheral circulation.^[^ ^23^ ^]^ Our results also demonstrated that BF‐ or BF EV‐transplantation promoted M2 polarization of macrophages in T2D mice (Figures 1 and 2) and that M2 polarization of peripheral monocytes/macrophages in patients with T2D VC was significantly increased (Figure 1). Importantly, the i*n vitro* experiments confirmed that BF EV‐induced M2 macrophages polarization enhanced the osteogenic differentiation of VSMCs (Figure 2). Regulation of immune cell activity is a critical mechanism by which intestinal bacteria influence disease progression. The bone marrow is the primary immune organ that serves as a significant target for the effects of intestinal bacteria.^[^ ^52^ ^]^ We found that BF EV accumulated in the bone marrow of T2D mice (Figure 2). Transplantation of T2D mouse bone marrow into wild‐type (WT) mice, vielded a significant increase in the number of peripheral M2 monocytes/macrophages, alongside an increase in aortic osteogenic differentiation, expecially miR‐32, which we first discovered has the function of promoting T2D vascular calcification, was significantly up‐regulated in the aorta.^[^ ^53^, ^54^ ^]^ However, no significant changes were observed in the intestinal BF (Figure S1, Supporting Information). This suggests that BF EV may exacerbate T2D VC by inducing M2 polarization of bone marrow macrophages.

To illustrate the importance of macrophages in T2D VC exacerbated by BF EV, the macrophages in T2D mice were cleared (Figure 3; Figure S5, Supporting Information). The results showed that the exacerbation of aortic calcification induced by BF EV was significantly inhibited. Flow cytometry analysis indicated a marked increase in total monocyte/macrophage count in the peripheral circulation; however, there was no significant change in the population of M2 macrophages. In contrast, although the total macrophage count in the bone marrow remained unchanged, there was a significant increase in the number of M2 macrophages was observed. Additionally, no significant change in serum Serpine1 levels were observed. This may be attributed to the fact that the bone marrow serves as the primary immune organ for macrophage development and that macrophages have a short renewal cycle. Consequently, when tissue macrophages are reduced in number, the differentiation of monocytes into macrophages is accelerated.^[^ ^55^, ^56^ ^]^ We found that macrophage populations continued to fluctuate during the clearance of chodronate liposomes and BF EV‐induced M2 polarization, resulting in an increase in bone marrow M2 macrophages; however, Serpine1 levels remained unchanged. This finding further supports the notion that the induction of M2 polarization in macrophages and the secretion of Serpine1 are crucial mechanisms through which BF EV promote vascular calcification in T2D.

Nucleic acids carried by EVs are crucial for regulating the biological activities of target cells.^[^ ^57^ ^]^ Sting is a key molecule that enables immune cells to respond to extracellular DNA stimulation and initiate immune responses.^[^ ^58^, ^59^ ^]^ Activated Sting often mediates inflammatory immune responses.^[^ ^60^ ^]^ However, we found that BF EV upregulated Sting expression in macrophages, and that the induced macrophages exhibited an anti‐inflammatory M2 phenotype (Figure 4). We speculate that BF EV‐mediated Sting activation likely induces macrophage M2 polarization through non‐classical pathways. Sting is primarily located in the endoplasmic reticulum, where it is induced and activated.^[^ ^61^ ^]^ Its location that coincides with that of Mef2d, a protein that has been extensively studied and that plays a significant role in the endoplasmic reticulum.^[^ ^62^ ^]^ Our investigations using co‐IP, in vivo co‐localization analysis, protein docking, and other technical methods revealed that Sting binds to Mef2d and induces its phosphorylation (Figure 4). Once activated, Mef2d translocates to the nucleus, where it exerts its transcriptional function.^[^ ^63^ ^]^ Furthermore, we found that Mef2d binds to the Trib1 promoter region, thereby promoting Trib1 transcription (Figures 4 and 5; Figure S2, Supporting Information). Trib1 serves as a positive regulator of macrophage M2 polarization^[^ ^64^ ^]^ and counteracts the inhibitory effect of si‐Mef2d on macrophage M2 polarization (Figure S4, Supporting Information). These findings demonstrate that BF EVs induce macrophage M2 polarization via the Sting‐Mef2d‐Trib1 axis.

Animal experiments have demonstrated that intragastric administration of BF or BF EV resulted in calcium ion deposition and osteogenic differentiation occurred in the aorta of T2D mice, with no evident presence of macrophage (Figures 1 and 2). This suggests that M2 macrophages may exacerbate T2D VC via the endocrine pathway. Further investigation revealed that Serpine1, a pro‐T2D VC cytokine, is secreted by BF EV‐activated M2 macrophages (Figures 5 and 6) and that its expression is regulated by Sting‐Mef2d signaling (Figure 5). Previous studies have established a positive correlation between Serpine1 and coronary artery calcification in patients with type 1 diabetes.^[^ ^65^ ^]^ Additionally, we found that Serpine1 was highly expressed in the peripheral blood of patients with T2D VC, and the constructed multiple logistic regression model we constructed showed a strong positive correlation with T2D VC (Figure 7; Table 3). These findings indicate that BF EVs induce elevated expression of Serpine1 in M2 macrophages via Sting‐Mef2d signaling, thereby contributing to the aggravation of T2D VC.

In conclusion, our study demonstrated that T2D‐related intestinal BF secrete EVs that promote macrophage M2 polarization and release Serpine1, which in turn facilitates VC. Clinical studies have further confirmed that peripheral blood M2 monocyte/macrophages and Serpine1 in the peripheral blood are two significant risk factors for T2D‐associated VC. This study provides a novel theoretical and experimental foundation for optimizing the prevention and treatment strategies for VC in patients with T2D.

## Conflict of Interest

The authors declare no conflict of interest.

## Author Contributions

C.C. conducted laboratory experiments, data analysis, and wrote the manuscript; animal feeding was carried out by Z.L. and Y.H.; portions of the experiments were performed by Y.G. and S.O.; manuscript review was undertaken by L.W.; and guidance on experimental design and manuscript review was provided by J.L. and C.J. All authors have read and approved the final manuscript.

## Supporting information



Supporting Information

## Data Availability

The data that support the findings of this study are available from the corresponding author upon reasonable request.
