# Supplementary material for: Bacteroides Fragilis Exacerbates T2D Vascular Calcification by Secreting Extracellular Vesicles to Induce M2 Macrophages
Source: Adv Sci (Weinh). 2024 Dec 12;12(5):2410495. doi: 10.1002/advs.202410495 (PMC11791993; doi:10.1002/advs.202410495)
Supplement: Supplementary file 1 — Supporting Information [file ADVS-12-2410495-s001.docx]

Supplementary Figure S1


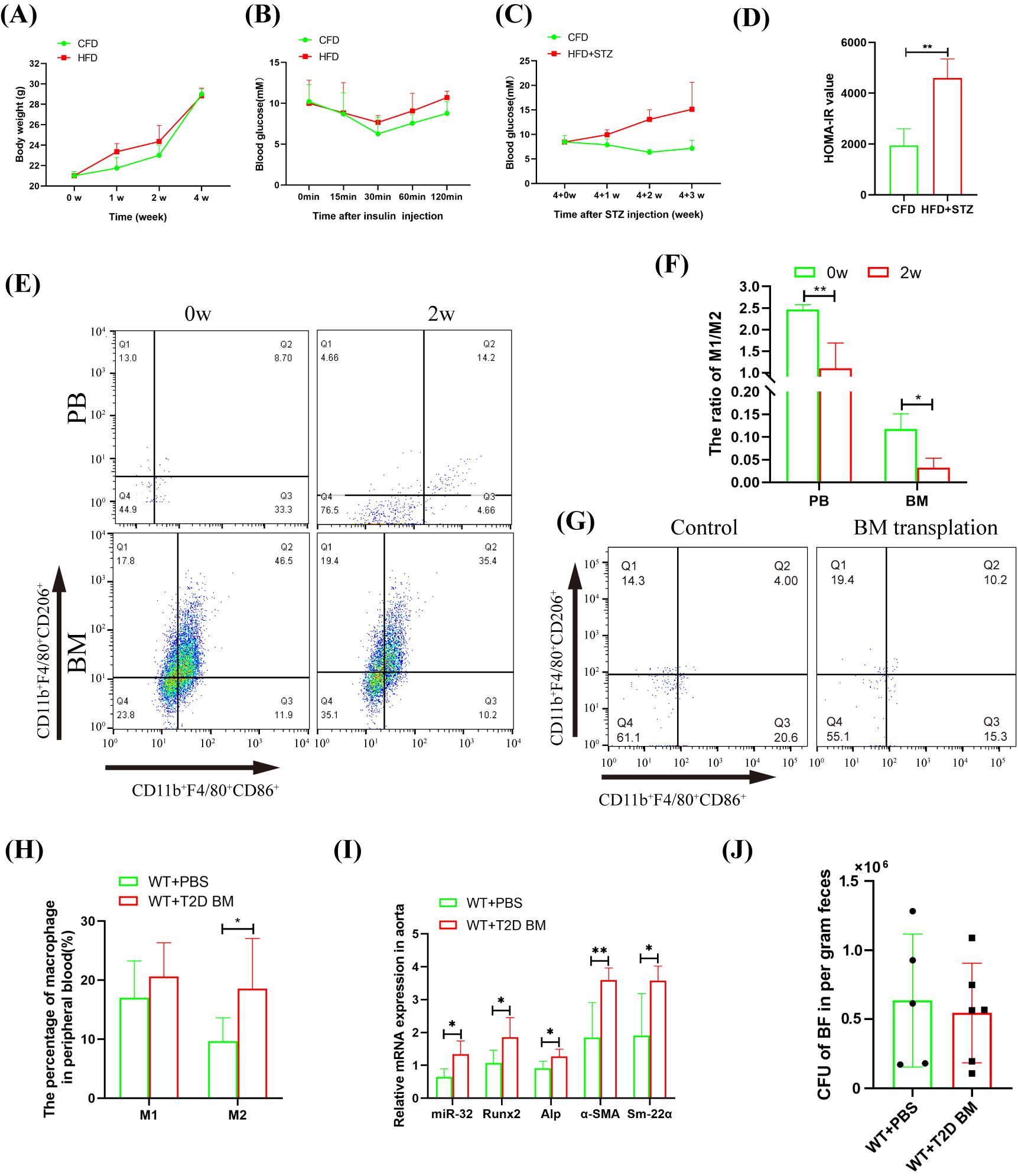


Supplementary Figure S1. Analysis of the causal relationship between BF and bone marrow macrophage polarization during the progression of T2D. **(A)** Body weight of T2D mice. **(B)** IPITT analysis. **(C)** Blood glucose analysis in T2D mice after modeling. **(D)** HOMA-IR analysis. **(E, F)** Flow cytometry analyzed monocytes/macrophage polarization in bone marrow and peripheral blood of T2D mice during disease progression (E), and statistical analysis (F). **(G, H)** Flow cytometric analyzed the effect of bone marrow transplantation from T2D mice to WT mice on the polarization of peripheral monocytes/macrophages (G), and statistical analysis (H). **(I)** qRT-PCR analyzed the effect of bone marrow transplantation from T2D mice to WT mice on the genes expression related to aortic osteogenic differentiation. **(J)** qRT-PCRanalyzed the effect of bone marrow transplantation from T2D mice to WT mice on the intestinal BF. Animal experiments included ≧5 mice, the experiment was repeated three times, the results are expressed as mean ± standard error, * means p < 0.05，**means p < 0.01.

Supplementary Figure S2


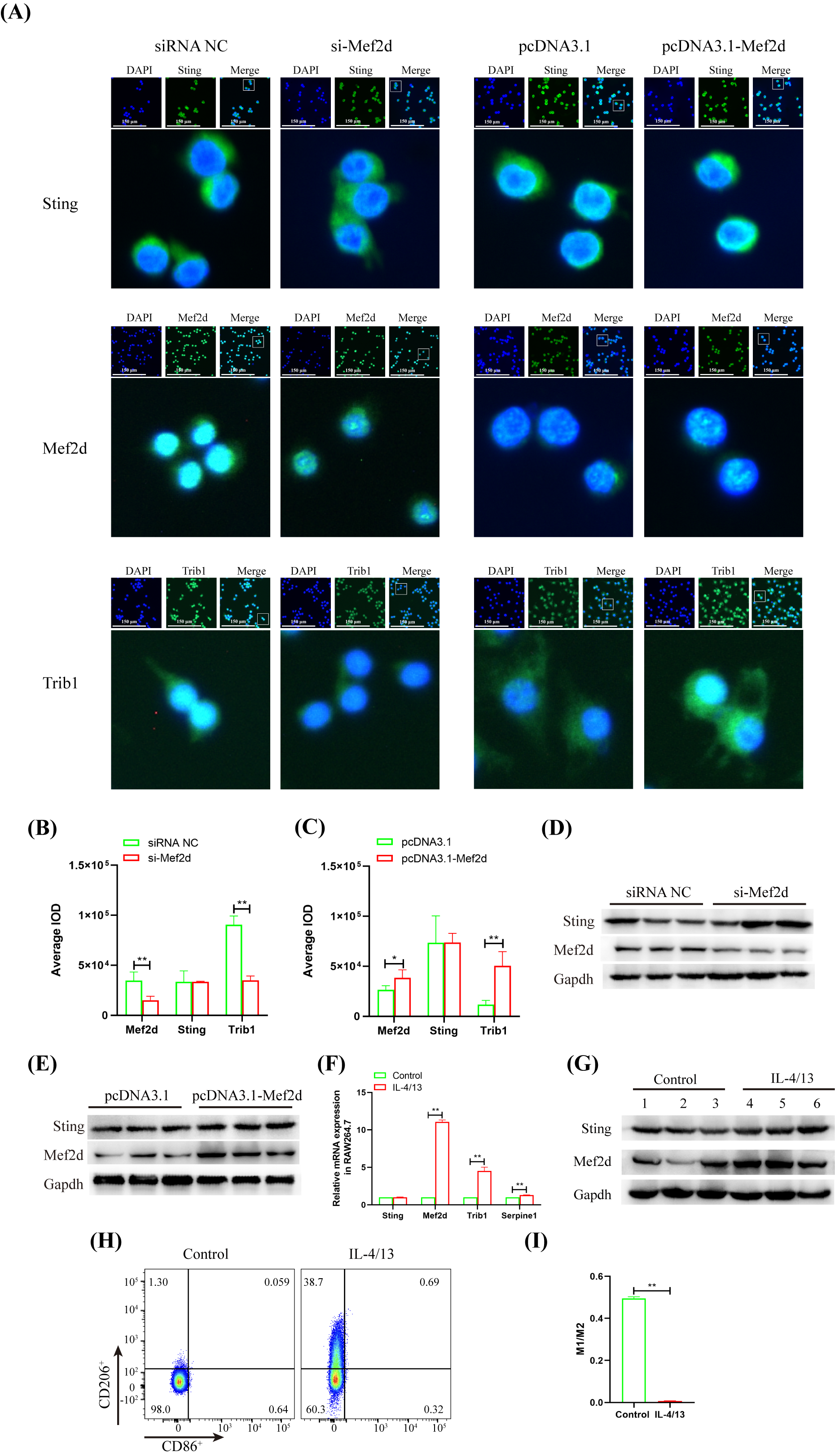


Supplementary Figure S2. Analysis of the relationship between Mef2d, Sting and macrophage M2 polarization. **(A-C)** Immunofluorescence analyzed the effect of si-Mef2d or pcDNA3.1-Mef2d transfection on the expression of Sting and Trib1 in macrophages (A), and statistical analysis (B, C). **(D)** Western blotting analyzed the effect of si-Mef2d transfection on the expression of Sting and Mef2d in macrophages. **(E)** Western blotting analyzed the effect of pcDNA3.1-Mef2d transfection on the expression of Sting and Mef2d in macrophages. **(F)** qRT-PCR analyzed the effect of IL-4/13-induced M2 macrophages on gene expression. **(G)** Western blotting analyzed the effect of IL-4/13-induced M2 macrophages on gene expression. **(H, I)** Flow cytometric analyzed the effect of IL-4/13 stimulation on M2 polarization of macrophages (H), and statistical analysis (I). The experiment was repeated three times, the results are expressed as mean ± standard error, * means p < 0.05，**means p < 0.01.

Supplementary Figure S3


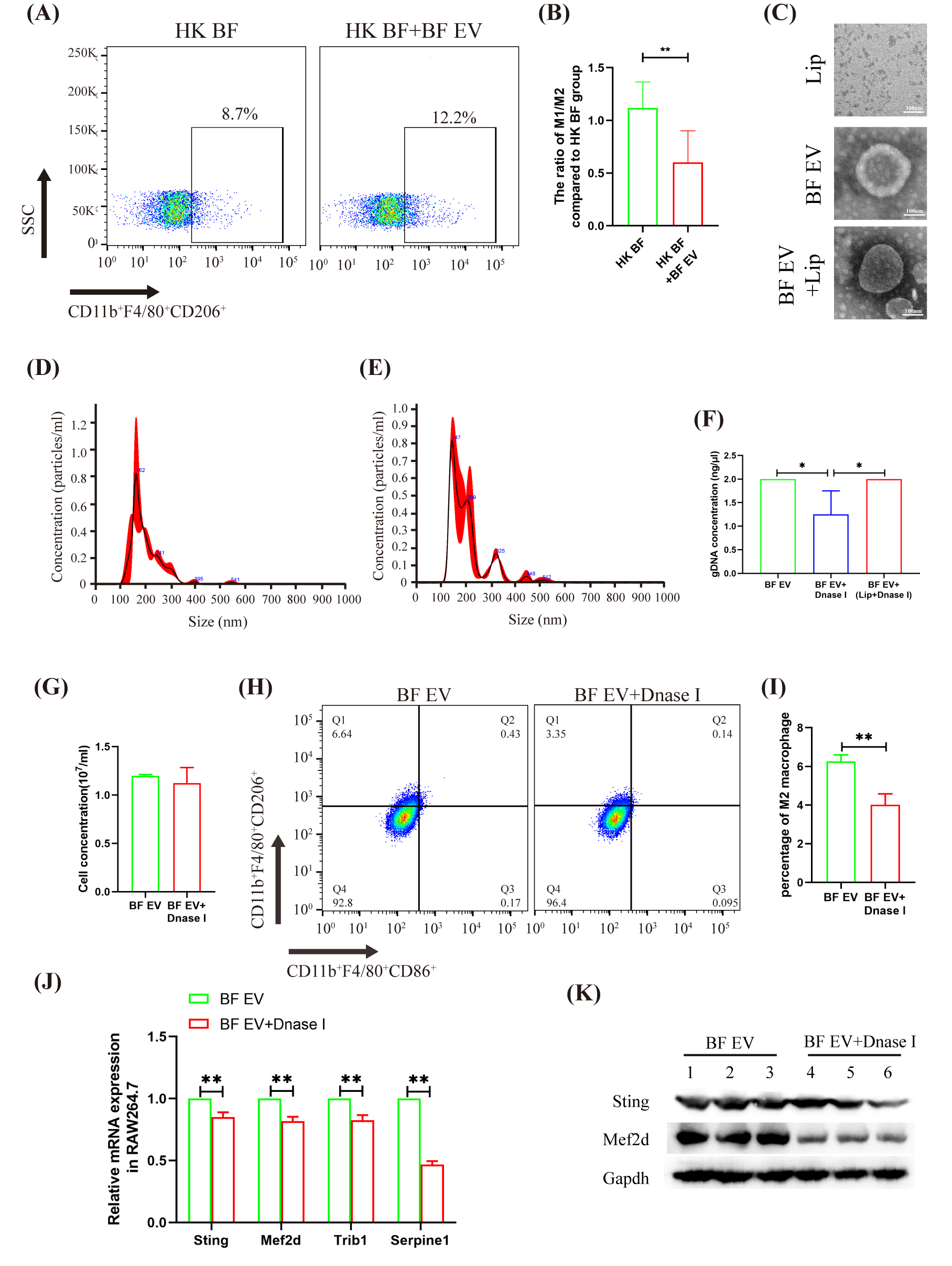


Supplementary Figure S3. Analysis of the mechanism by which BF EV promotes macrophages M2 polarization . **(A，B)** Flow cytometric analyzed the effect of BF EV transplantation on M2 polarization of mouse bone marrow macrophages (A), and statistical analysis (B). **(C)** Transmission electron microscope observation of recombinant BF EV morphology. **(D，E)** NTA analysis of normal BF EV and recombinant BF EV particle size. **(F)** Analysis of DNA concentration in Dnase I recombinant BF EVs or Dnase I-treated BF EVs. **(G)** Counting analysis of the effect of Dnase I-treated BF EV on macrophages. **(H，I)** Flow cytometric analyzed the effect of Dnase I-treated BF EV on macrophage polarization (H), and statistical analysis (I). **(J)** qRT-PCR analyzed the effect of Dnase I-treated BF EV on macrophage gene expression. **(K)** Western blotting analyzed the effect of Dnase I-treated BF EV on macrophage gene expression. The experiment was repeated three times, the results are expressed as mean ± standard error, * means p < 0.05，**means p < 0.01.

Supplementary Figure S4


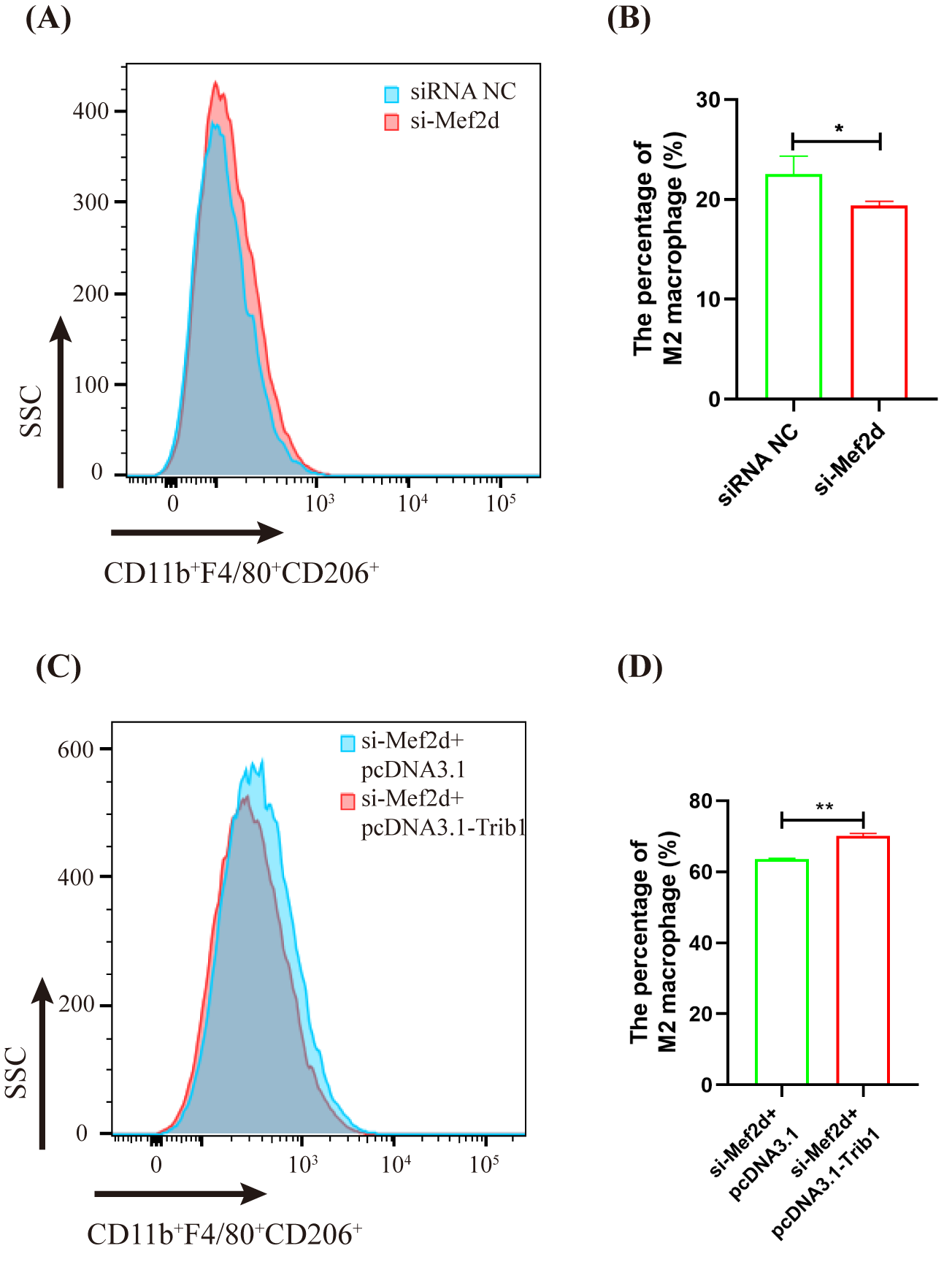


Supplementary Figure S4. Rescue experiment analyzed the relationship between Trib1 and Mef2d. **(A，B)** Flow cytometry analyzed the effect of si-Mef2d transfection on macrophage M2 polarization (A), and statistical analysis (B). **(C，D)** Flow cytometry analyzed the effect of co-transfection of pcDNA3.1-Trib1 and si-Mef2d on macrophages M2 polarization (C), and statistical analysis (D). The experiment was repeated three times, the results are expressed as mean ± standard error, * means p < 0.05，**means p < 0.01.

Supplementary Figure S5


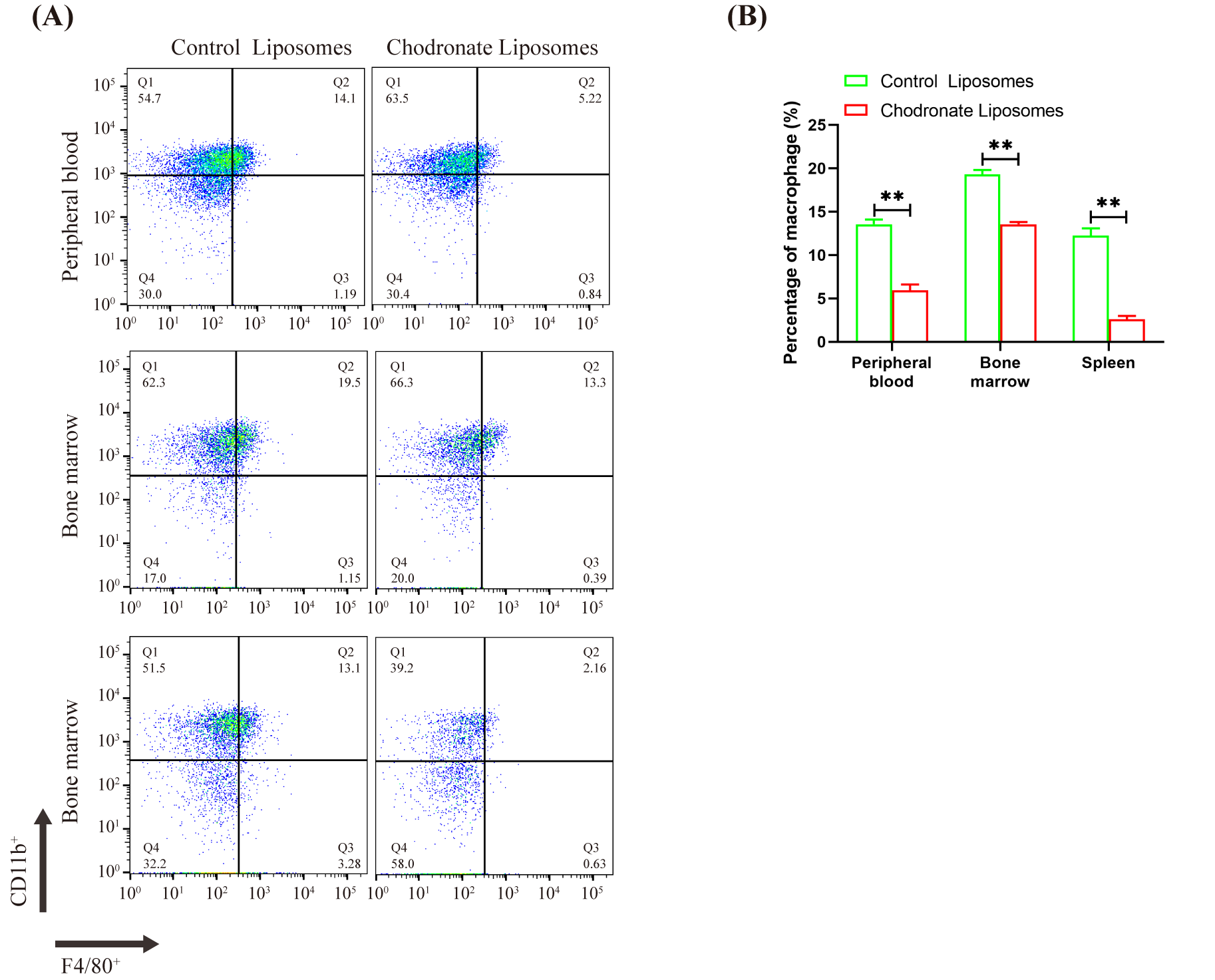


Supplementary Figure S5. Analysis of the clearance efficiency of Chodronate Liposomes on mice macrophages. **(A，B)** Flow cytometry analyzed macrophages in peripheral blood, bone marrow and spleen (A), and statistical analysis (B). Animal experiments included =3 mice, the experiment was repeated three times, the results are expressed as mean ± standard error, * means p < 0.05，**means p < 0.01.

**Supplementary Table**

Supplementary Table S1. Primers sequence

| Genes | species | primers | Sequence(5’→3’) |
| --- | --- | --- | --- |
| Runx2 | Mouse | Forward | TGTCCGCCACCACTCACTACC |
|  |  | Reverse | GGGAACTGATAGGATGCTGACGAAG |
| Alp | Mouse | Forward | CACGGCGTCCATGAGCAGAAC |
|  |  | Reverse | CAGGCACAGTGGTCAAGGTTGG |
| αSma | Mouse | Forward | CGTGGCTATTCCTTCGTGACTACTG |
|  |  | Reverse | CGTCAGGCAGTTCGTAGCTCTTC |
| Sm22α | Mouse | Forward | ACTCTAATGGCTTTGGGCAGTTTGG |
|  |  | Reverse | CCTCTTATGCTCCTGGGCTTTCTTC |
| miR-32 | Mouse | Forward | CGCGCTATTGCACATTACTAAGTTGCA |
| Sting | Mouse | Forward | AAGGAGGAGGTTACCATGAATGCC |
|  |  | Reverse | CAGATGAGGTCAGTGCGGAGTG |
| Mef2d | Mouse | Forward | GGCTGGCACTAGGCAATGTCAC |
|  |  | Reverse | CTGCTGTGGCTGTGGCTGTG |
| Trib1 | Mouse | Forward | TGCCTCATCCGAAGCCTCCTAAG |
|  |  | Reverse | GAGTCAACATAGCCCGGTTCCAAG |
| Serpine1 | Mouse | Forward | GTGCTGGTGAATGCCCTCTACTTC |
|  |  | Reverse | GACGGTGCTGCCATCAGACTTG |
| Gapdh | Mouse | Forward | TGTTTCCTCGTCCCGTAG |
|  |  | Reverse | CAATCTCCACTTTGCCACT |
| MEF2D | Human | Forward | TCTGATTTGGTCGTATTGGG |
|  |  | Reverse | TTTAGGGTAGTGGTAGAAGG |
| STING | Human | Forward | CCTGATAACCTGAGTATGGCTGACC |
|  |  | Reverse | GTTGCTGTAAACCCGATCCTTGATG |
| TRIB1 | Human | Forward | TCATTCGCAGCCTCTTGAGACG |
|  |  | Reverse | TCACTGTCCTCCTGGTACTCTGG |
| SERPINE1 | Human | Forward | ACGGCTGGTGCTGGTGAATG |
|  |  | Reverse | CGGGCGTGGTGAACTCAGTATAG |
| GAPDH | Human | Forward | TCTGATTTGGTCGTATTGGG |
|  |  | Reverse | TTTAGGGTAGTGGTAGAAGG |
| B. fragilis | *Bacteroides fragilis* | Forward | TGGACTGCAACTGACACTGA |
|  |  | Reverse | GCCGCTTACTGTATATCGCA |

Supplementary Table S2. Antibodies used in the experiments

| Antibody name | Source | Manufacturer | Catalog # (RRID) | Application |
| --- | --- | --- | --- | --- |
| Anti-Mef2d | Rabbit | Proteintech | 14353-1-AP#AB_2878046 | WB(1:1000)  IF(1:100) |
| Anti-Sting |  |  |  |  |
| Anti-Trib1 |  |  |  |  |
| Anti-CD206 | Goat | R&D Systems | AF2535# | IF(1:100) |
| Anti-phospho-Mef2d |  |  |  |  |
| Anti-Serpine1 |  |  |  |  |
| Anti-ɑSMA | Rabbit | Cell signaling | 19245#AB_2734735 | WB(1:1000) |
| Anti-Runx2 | Rabbit | abcam | 20700-1-AP#AB_2722783 | WB(1:500) |
| Anti-Sm22ɑ | Rabbit | Abcam | 40471#AB_443021 | WB(1:1000) |
| Anti-Alp | Rabbit | Proteintech | 11187-1-AP#AB_2305523 | WB(1:1000) |
| Anti-F4/80-APC | Rat | Thermo Fisher Scientific | 17-4801-80#AB_2784647 | IF(1:50) |
| Anti-CD11b-Percp-Cy5.5 | Rat | Thermo Fisher Scientific | 45-0112-80#AB_953560 | IF(1:50) |
| Anti-CD86-FITC | Rat | Thermo Fisher Scientific | 11-0862-82#AB_465148 | IF(1:50) |
| Anti-CD45-FITC | Mouse | BD Pharmingen | 560976#AB_395874 | FC (1:100) |
| Anti-CD11b PERCP-Cy5.5 | Rat | BD Pharmingen | 561114#AB_394002 | FC (1:100) |
| Hu CD206 APC | Mouse | BD Pharmingen | 561763#AB_398476 | FC (1:100) |
| Anti-CD68-PE | Mouse | BD Pharmingen | 556059#AB_2739021 | FC (1:100) |

Supplementary Table S3. Patients clinic datas

|  | T2D | T2D VC | Correlation analyse | |
| --- | --- | --- | --- | --- |
|  |  |  | Pearson correlation | *p* |
| Male/Femal | 30/13 | 26/27 | -0.21 | 0.02**^∗^** |
| Ages | 51.1±11.7 | 57.5±7.1 | 0.33 | <0.01**^∗^** |
| Number of monocytes | 0.48±0.24 | 0.43±0.14 | -0.13 | 0.10 |
| Percentage of monocytes | 6.82±2.32 | 6.83±1.94 | 0.00 | 0.49 |
| Phosphorus | 1.01±0.19 | 1.11±0.24 | 0.09 | 0.20 |
| calcium | 2.27±0.12 | 2.25±0.14 | -0.09 | 0.21 |
| Glycohemoglobin (%) | 10.10±2.41 | 10.15±2.46 | 0.01 | 0.45 |
| Blood glucose | 9.14±3.87 | 9.94±5.14 | 0.08 | 0.23 |
| Total cholesterol | 4.83±1.23 | 4.81±1.07 | -0.02 | 0.43 |
| Triglycerides | 3.34±3.72 | 2.32±1.56 | -0.19 | 0.03**^∗^** |
| HDL | 1.00±0.30 | 1.16±0.46 | 0.19 | 0.03**^∗^** |
| LDL | 2.67±0.90 | 2.87±1.01 | 0.10 | 0.17 |
| Apolipoprotein A1 | 1.14±0.21 | 1.20±0.26 | 0.20 | 0.03**^∗^** |
| Apolipoprotein B | 0.99±0.54 | 1.09±0.37 | 0.08 | 0.22 |

# Note: Significance difference is labeled with one star (*p*<0.05).
